# Isolated during adolescence: long-term impact on social behavior, pain sensitivity, and the oxytocin system in male and female rats

**DOI:** 10.1186/s13293-024-00655-7

**Published:** 2024-10-15

**Authors:** Akseli P. Graf, Anita C. Hansson, Rainer Spanagel

**Affiliations:** Institute of Psychopharmacology, Medical Faculty Mannheim, Central Institute of Mental Health, University of Heidelberg, J5, 68159 Mannheim, Germany

**Keywords:** Social isolation, Post-weaning social isolation, Sex differences, Social memory, Oxytocin, Paraventricular Nucleus of the Thalamus (PVT), Paraventricular Nucleus of the Hypothalamus (PVN)

## Abstract

**Background:**

Adolescent social isolation (ASI) has profound long-term effects on behavioral and neural development. Despite this, the specific long-term impact of ASI during different adolescent stages and across sexes remain underexplored.

**Methods:**

Our study addresses this gap by examining the effects of early- and late- adolescent social isolation on both male and female rats. Rats were either isolated (or group-housed) starting from PD 21 (early) or PD 42 (late) for three weeks and then rehoused into groups. In adulthood (PD 90), rats underwent a battery of tests: elevated plus-maze, open field, novel object recognition, social interaction and social recognition memory and hotplate tests. Finally, we analyzed oxytocin receptor binding in several regions in the brains of a second cohort of rats.

**Results:**

Both, male and female rats from the late adolescent social isolation (LASI) groups spent significantly less time interacting in the social interaction test. Additionally, we observed a general decrease in social recognition memory regardless of sex. Both male ASI groups demonstrated heightened thermal pain sensitivity, while the opposite was observed in early adolescent social isolation (EASI) female rats. In the brain, we observed changes in oxytocin receptor (OTR) binding in the paraventricular nucleus of the hypothalamus (PVN) and paraventricular nucleus of the thalamus (PVT) and central amygdala (CeA) with the largest changes in EASI and LASI female rats.

**Conclusion:**

Our model demonstrates long-lasting alterations on behavior and oxytocin receptor binding levels following ASI providing insights into the long-term effects of ASI in a time- and sex-specific manner.

**Supplementary Information:**

The online version contains supplementary material available at 10.1186/s13293-024-00655-7.

## Highlights

- **Social Interaction Reduced**: Regardless of the timing of social isolation, male rats displayed heightened sensitivity to thermal pain, while only the early social isolation altered pain sensitivity in females.
- **Impaired Social Recognition Memory**: Isolation caused a general impairment in recognizing previously encountered rats, which was specific to social encounters but not objects. Suggesting the deficit is domain specific.
- **Alterations in Pain Sensitivity**: Rats isolated during late adolescence showed significantly less social interaction compared to those raised in groups.
- **Significant Brain Changes in Females**: Female rats isolated during adolescence exhibited a substantial increase in oxytocin receptor binding in key brain regions (PVT, PVN ) which are important for social behavior and anxiety.

**Supplementary Information:**

The online version contains supplementary material available at 10.1186/s13293-024-00655-7.

## Plain language summary

Our study explored whether the timing of being socially isolated during adolescence affected anxiety, social and pain-related behaviors in adult rats. We were also interested in understanding whether there were any sex differences between socially isolated female and male rats if isolated at different periods throughout adolescence. We had two isolation groups, in one rats were kept isolated during a time point in their life that represents early adolescence and the other group during late adolescence. These groups were compared to a control group of rats that remained group housed throughout the study. In adulthood, we tested all groups in a battery of behavioral tests and collected their brains for further investigation. In the brain, we examined the oxytocin system for changes in key regions involved in anxiety and social behavior and found changes in the thalamus, hypothalamus and amygdala.

Our main findings were

• Rats that were isolated during their late adolescent years interacted less with other rats.

• All social isolated rats had trouble recognizing other rats they had previously met.

• Male rats isolated at any time point in adolescence were more sensitize to heat pain, while females isolated early in adolescence showed reduced sensitivity to heat pain.

• We observed sex-specific changes in the oxytocin receptor (a neuropeptide related to social and emotional behaviors) binding in the paraventricular nucleus of the hypothalamus, paraventricular thalamus, and the central amygdala.

In conclusion, our study suggests that social isolation during adolescent years can have lasting effects on behavior, particularly in social interactions, social memory and sensitivity to pain as well as altering the oxytocin system.

## Introduction

Adolescence is a period [1, 2] that is vital for fostering emotional bonding and physical growth, central for future well-being and development in most mammals [3]. During adolescence, bonding [4], playing [5] and social learning [6] are all important features of normal development, which require social interactions in both rats and humans alike. Adolescents in general spend more time with their peers and show greater willingness for risk-taking and sensation seeking [7].

On the flipside, adolescence is also a period when humans and rodents alike show higher stress reactivity [8, 9]. Exposure to adolescent adversity can have long-lasting effects by rewiring critical neural pathways and these changes can become “biologically embedded” [10]. In humans this is further emphasised by the significant increase in emergence of neuropsychiatric disorders during adolescence, whereby 50% of all lifetime occurrences emerge by age 14 [11] with significant differences in the presentation of disorders across sexes [12]. Further suggesting that adolescence is a critical developmental period with a heightened vulnerability to adversity and stress.

Recently, a type of adversity that affected us all was the COVID-19 pandemic. It’s social isolation measures emphasized the importance of in-person social interactions on both physical and mental health [13]. The social isolation measures during the pandemic had a particularly negative consequences on adolescents’ development and mental health [8, 14]. The scope and persistence of the negative effects of adolescent social isolation remain unclear. Hence, studying the long-term effects of adolescent social isolation (ASI) is an important societal question. Here, the use of preclinical models allows us to control and study the longitudinal impact of ASI on both brain and behavior to a degree not possible in humans.

The approach we used here was to isolate male and female rats during early and late adolescence (see methods). After the isolation period we re-socialized them back into groups before testing them in a battery of behavioral tests in adulthood 1. In the ASI paradigm, rats are individually housed in their home cage with water and food but lack somatosensory contact, but still had olfactory, auditory, and visual stimulation from other rats in the colony room. This model demonstrates good face validity, as isolated humans typically have visual, olfactory and auditory stimulation from their surroundings (i.e. smart phones, digital media) but often lack social touch or contact [15].

Since previous ASI studies in rats have observed lasting changes in the social domain; e.g. ASI reduces social approach [16] and social interaction [17, 18] we hypothesized that ASI will lead to impairments in social behaviors and alterations in the oxytocin (OT) system. Our hypothesis builds on evidence that the OT system is modulated at least in the short-term in response to adolescent adversity [1, 19, 20]. However, the long-term effects of adolescent adversities on the OT system remain largely unexplored and to our knowledge, no studies have investigated the long-term effects of adolescent social isolation on the OT system in rats (See meta-analysis by Krimberg et al. [21]). OT receptors peak around PD21 and reach adult levels between PD 56–84 depending on strain [22, 23]. These data demonstrate how the OT system undergoes changes during adolescence, and a lack of social stimulus during this period could significantly alter the developmental trajectory of the OT system to adapt to a low stimuli environment.

The long-term effects of timing and sex-differences of ASI on the brain and behavior remain poorly understood. Here we aimed to characterize both the effect of early ASI (EASI) (PD 21–42) and late ASI (LASI) (PD 42–63) and their potential sex-specific effects on social behaviors, memory, thermal pain and anxiety-like behaviors in adulthood. We chose these periods for two reasons. First, EASI and LASI coincide with a pre-pubertal and post-pubertal phase in our Wistar rats. Allowing us to investigate how puberty can influence behavior [24, 25]. Second, the gradual decline in OTR density from PD21 until about PD60 could suggest that the EASI and LASI period could see different alterations in OTR binding which would in turn influence behavior in a different manner [23]. Furthermore, we characterized the molecular sequelae of adolescent social isolation on OTR bindings in key regions associated with the above-mentioned behavioral domains. These included paraventricular nucleus (PVN) of the hypothalamus, central and basolateral amygdala (AMY), and the paraventricular nucleus of the thalamus (PVT). The aforementioned paraventricular structures are midline structure that has recently garnered significant interest due to their high expression of OTRs [23, 23], OTR modulation following adversity (social defeat) [27], and for its involvement in a wide array of behavioral processes linked to other early adversities [28] making them prime targets for investigation following ASI.

## Methods

### Animals and housing

Male (*n* = 40) and female (*n* = 40) outbred WIST: RccHan rats were purchased from Envigo (Venray, Netherlands) and arrived at the institute on (PD 21). These rats were used to characterize the behavioral sequelae of ASI in adulthood. A separate cohort of male (*n* = 24) and female (*n* = 24) rats were purchased from the same supplier (that also arrived on PD 21) and used for characterizing the molecular of OTRs. Rats were housed individually (Makrolon Type III cages) or in groups of four (Makrolon Type IV cages) under a standard diurnal 12 h light-dark cycle, temperature 23 ± 3, and humidity (40–60%) with free availability of tap water and standard laboratory chow without any enrichment. Male and female rats were housed in separate colony rooms. All experiments were approved by the local animal care committee (Regierungspräsidium Karlsruhe, Referat 35, Karlsruhe, Germany, AZ35-9185.81/G-289/18) following the guidelines of the European Union (2010/63/EU).

### Study design

**Fig. 1:**
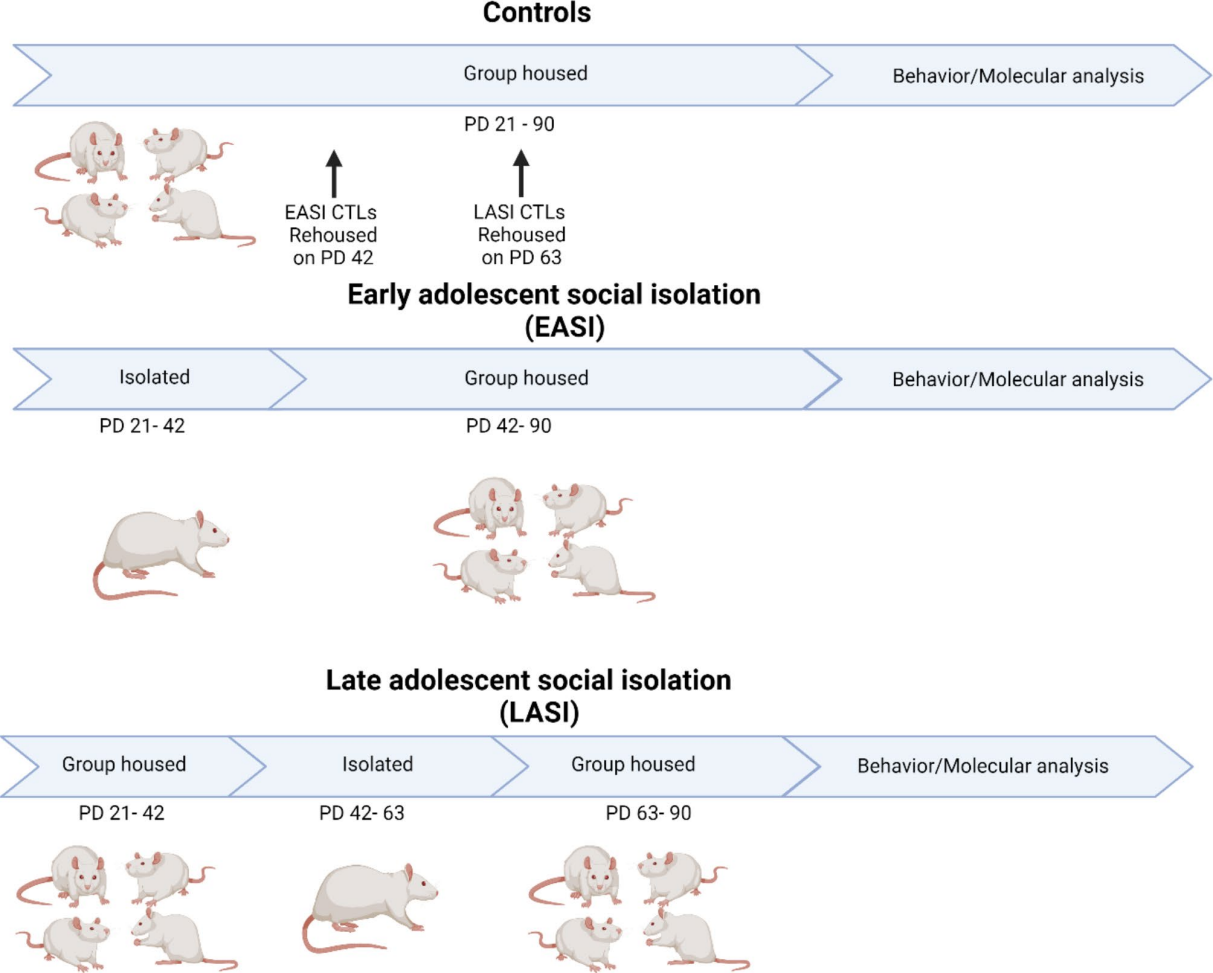
Timeline of adolescent social isolation procedures. EASI (PD 21–42) and LASI (PD42-63) rats underwent three weeks of social isolation and were then rehoused with rats from the same condition, whereas control rats remained group-housed throughout the study. In the first experiment, the rats underwent behavioral testing starting at PD 90. In the second experiment, rats were sacrificed on PD 90 for comparison. The timeline was created with BioRender.com

All rats were weaned on PD 21 and were pseudo-randomly selected for housing into either the early adolescent social isolation (EASI), late adolescent social isolation (LASI) condition or control (CTL) condition. Rats were housed in groups of four rats per cage. Each isolation condition lasted for three weeks. Two cohorts of rats where used in the behavioral study, in order to handle the large amount of rats used in the study. In the first cohort, we used eight control rats, eight EASI and four LASI rats of each sex. In the second cohort, we used eight control rats, four EASI, and eight LASI rats of each sex. In the EASI condition, the rats were socially isolated from PD 21 to 42, and in the LASI condition, the rats were socially isolated from PD 42 to 63 (Fig. 1). For the duration of the social isolation, rats had no somatosensory contact but had olfactory, auditory, and visual stimuli of the other rats in the same colony room from the same and different conditions. At the end of the isolation period rats were rehoused with rats from the same condition. Simultaneously, control rats were rehoused with other control rats to equalize potential rehousing stress among groups (Fig. 1). Rats remained group housed for the remainder of the experiment.

**Fig. 2:**
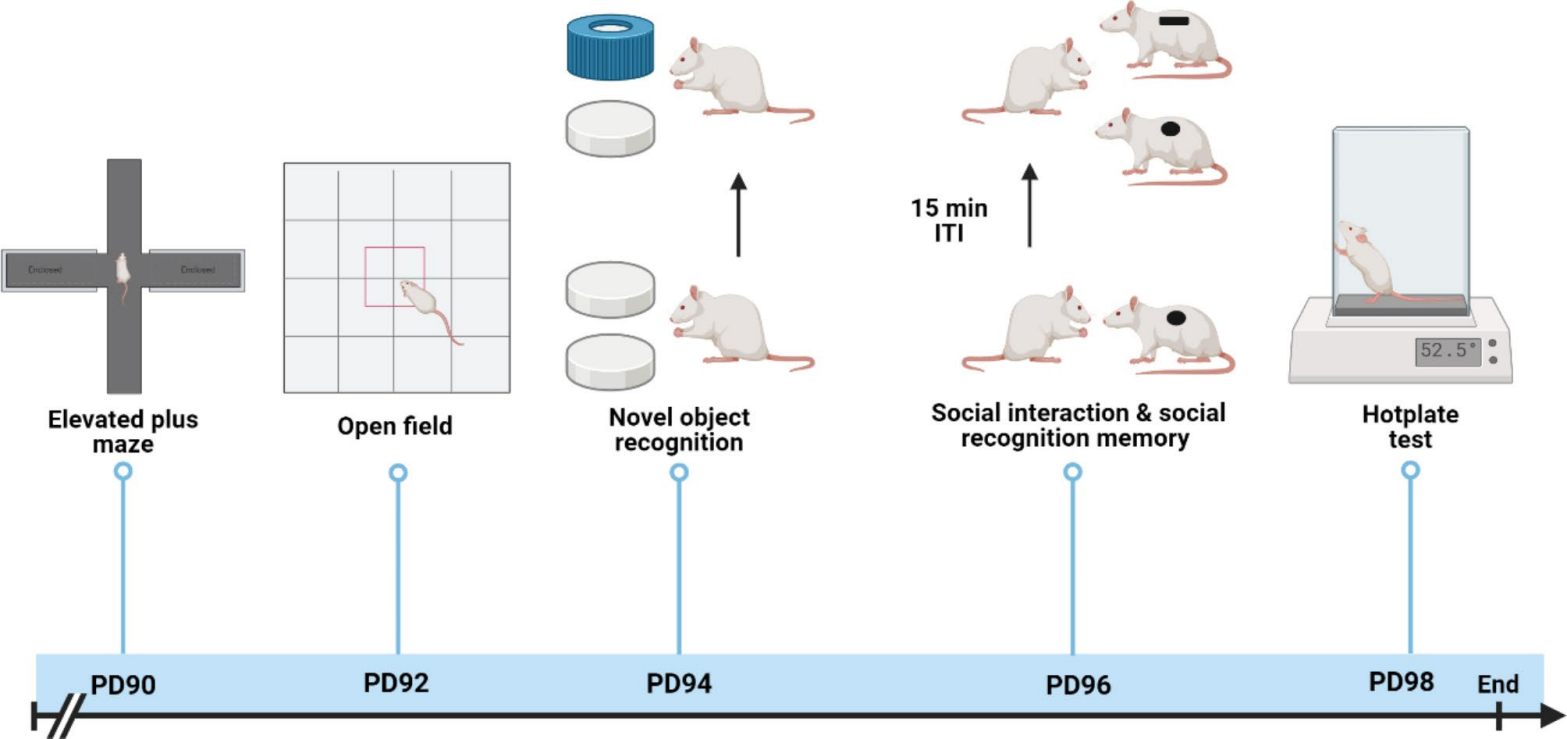
Timeline for behavioral experiments. Behavioral experiments were performed in adulthood for EASI, LASI, and CTL rats starting from PD 90 with elevated plus maze (EPM), open field test (OFT), novel object recognition (NOR), social interaction test (SIT), social recognition memory (SRM), and hotplate test. Created by BioRender.com

Behavioral testing began with the elevated plus maze (EPM) (PD 90), followed by the open field test (OFT) (PD 92), novel object recognition (NOR) (PD 94), social interaction and social recognition memory (SIT/SRM) (PD 96), and Hotplate test (PD 98) (Fig. 2). All behavioral testing was done during the first five hours to the light-ON cycle (inactive). A separate cohort of rats was used for the molecular characterization of OTR alterations in adulthood following ASI. These rats underwent the same ASI procedure as in experiment 1 but did not undergo behavioral testing. Instead, the rats were sacrificed on PD90 within the first two hours of the light-ON cycle.

Figure 2 Timeline for behavioral experiments. Behavioral experiments were performed in adulthood for EASI, LASI, and CTL rats starting from PD 90 with elevated plus maze (EPM), open field test (OFT), novel object recognition (NOR), social interaction test (SIT), social recognition memory (SRM), and hotplate test. Created by BioRender.com.

### Behavioral tests

We chose commonly used behavioral tests to assess for anxiety-like, social and memory processes as well as pain sensitivity that had previously been shown to be altered by social isolation and had already been validated in the lab. All behavioral tests were performed during the first five hours of the inactive phase (light ON) of the diurnal cycle. Rats were given at least 48 h rest between tests. All videos were recorded and evaluated offline by an expert blinded to the experimental manipulations. The estrous cycle of females was tracked after the elevated plus-maze (EPM) and hotplate test (HP) as there were indications that anxiety-like behaviors [29] and thermal pain sensitivity [30, 31] are influenced by the estrous cycle. All behavioral apparatuses were cleaned with 70% alcohol solution at the start of each day, between trials, and after each day of testing to prevent the transmission of olfactory cues. Next, the apparatuses were cleaned with water and allowed to dry as evidence suggests that strong scented solutions like alcohol can influence behavioral results [32].

### Estrous cycle cytology

Cytological vaginal smears were collected immediately after the elevated plus-maze and hotplate test to monitor the estrous cycle phase, as evidence points to the estrous cycle phase influencing both anxiety-like behavior and pain sensitivity in these two tests [29, 30]. The samples were analyzed under a light microscope (V300, Will Wetzlar) and characterized into two categories estrus/diestrus and proestrus/metestrus groups, where pain sensitivity differences appeared in the literature.

### Elevated plus maze

To measure anxiety-like behaviors, we used the EPM, which is an apparatus shaped like a plus sign made of dark gray PVC. It has two open arms measuring 50 cm × 12 cm each and two enclosed arms measuring 50 cm × 12 cm × 50 cm each that surround a middle platform measuring 12 cm × 12 cm, 50 cm above the floor. At the beginning of each trial, a rat was gently placed on the middle platform facing an open arm and then allowed to explore the EPM (90 lx) for 5 min. The subsequent video analysis assessed the time spent in the open and closed arms, center time, number of entries made into the open or closed arms (where an entry was defined as all four paws in a particular arm), head dips, and risk assessment. Risk assessment was defined as the act of placing only the head or forepaws in the open arm without any accompanying movement of the hind legs, even if the rat subsequently entered the arm. The percentage of time spent in the open arms was calculated using the following formula: open arm time / (center + open arm + closed arm time) × 100. While center time was calculated using: center time / total time (center + open arm + closed arm time) × 100.

### Open field test

To assess the locomotor activity of the animals, we used the open field test, which measures the movement of test rats [33]. The apparatus comprised four uniformly sized arenas, each measuring 50 cm × 50 cm × 50 cm and was constructed from dark gray PVC. One day before testing, the rats were habituated to the experimental room for 15 min. On the test day, the rats were brought into the experimental room and habituated for 5 min before the test started. The rats were gently placed in the center of the arena facing a random side, and locomotor activity was measured during a 30-minute test (50 lx). The distance travelled in the OFT was measured in centimeters.

### Novel object recognition test

To assess object recognition memory in rats, we employed a test that comprised two phases, namely the initial 5 min acquisition phase (P1) and the 3 min test phase (P2), separated by an inter-trial interval (ITI) of 15 min. The rats were habituated to the open field for 15 min one day prior to testing. The objects under investigation were made of ceramics or glass. To ensure the accuracy of the test results, all objects and the test arena were thoroughly cleaned and dried with 70% ethanol before and during the test. We have conducted preliminary tests in our laboratory to find equally attractive to the subjects (approximately 50% preference) (data not shown) and used these for the test (see Supplementary materials). During P1, the rat was placed in the center of the open field and exposed to two identical unknown objects (A), after which the rat was returned to its home cage and the objects were cleaned and dried. In P2, the rat was returned to the open field and presented with the familiar object A′ (an identical copy of the object presented in P1) and a novel test object (B). An image of the objects can be found in Appendix 3. The duration of object exploration (sniffing, touching an object with whiskers, and licking) was recorded for both P1 and P2. The discrimination between the exploration time of the novel object and the familiar object was expressed as a percentage of the total exploration time of both objects during P2 [100/(A′+B) × B], whereas the discrimination index was calculated by subtracting the exploration time of the familiar object A′ from the novel object B in P2 (B − A′).

## Social interaction and recognition memory

To evaluate social interactions and social recognition memory (SRM) in rats, we utilized an experimental design, as described previously [34]. The test involved exposing the experimental rat to an unfamiliar young adolescent same-sex social partner (5–6 weeks old) for a duration of 5 min in the open field. No habituation was required, as the rats had already been exposed to the open field across the OFT and NOR. The experimental rat was placed in the open field and allowed to explore for 1 min, after which the stimulus rat was placed in the open field, and the 5 min SIT test began. The frequency of various social behaviors, including contact behavior such as social exploration including anogenital and non-anogenital investigations, were quantified for only the experimental rat. Additionally, the frequency of rearing and self-grooming was recorded.

In the second part of the test assessing social recognition memory, the initial 5-minute social interaction period with the unfamiliar social partner (A) served as the sample phase (P1) for the social recognition test (P2). In the subsequent test for social recognition memory, a second unfamiliar adolescent of the same sex (B) was introduced during the test (P2) after a 15-minute inter-trial interval. During P2, the familiar (A’) and novel social partners (B) were presented to the experimental animal for 3 min, and the time for social investigation (anogenital, non-anogenital exploration, and approach/following) for the test rat was recorded. To calculate the social discrimination percentage, we used a within-subjects design, where we calculated the exploration time of the novel conspecific expressed as a percentage of the total exploration time of both conspecifics during P2 [100/(A’+B) × B].

### Thermal pain sensitivity

Thermal pain sensitivity was quantified using a hot plate apparatus (Ugo Basil, New Jersey, USA) with a fixed temperature of 52.5 °C ± 0.1 °C. This experimental setup was conducted in accordance with the methods established previously [35], and video recording of the behavior was analyzed offline frame-by-frame. The experiment was performed in the colony room of the experimental rats to reduce potential environmental stress-induced analgesia [36]. In short, rats were gently placed onto the hotplate platform at the beginning of the experiment when the hotplate was at 52.5 °C, and the test was terminated as soon as the rat showed the first heat-provoked reaction or after a cut-off period of 30 s to avoid tissue damage (which no rat reached). The first heat-evoked responses, including foot shake, stamping, paw licking, or jumping off the platform, which were used as a cut-off measure of pain.

### Tissue collection and preparation

Rat brains were collected within the first two hours of the start of the inactive cycle. The rats were first dazed and then quickly and painlessly decapitated using a guillotine. The brains were quickly but carefully removed from the skull and flash frozen in 2-Methylbutane (-40 °C) until completely frozen (~ 20–40 s) and stored at -80 °C until further processing.

### Brain section preparation

To prepare the flash-frozen brains for sectioning, they were first removed from the freezer (-80 °C) and placed in a cryostat-microtome (~ -20 °C) (Leica CM 1950, Leica Biosystems) for 1 h for acclimatization prior to sectioning. After acclimatization, frozen brains were embedded in the specimen stage using O.C.T™ (Tissue-Tek) compound consisting of water-soluble glycols and resins. The brains were sectioned into 12 μm slices using a sharp blade, and brain sections were collected from the brain regions of interest using stereotaxic coordinates [37]. Brain sections from the following Bregma levels were collected; medial prefrontal cortex, Bregma: +3.20 to + 2.20, Nucleus accumbens shell and, Bregma: +1.70 to + 1.00, PVN), Bregma: amygdala, PVT, Bregma: -2.12 to -3.2), and Ventral tegmental area, Bregma: -5.2 to -6.00) (Appendix 7). Slices were collected and embedded onto gelatin-coated SuperFrost Plus slides (Thermo Fisher Scientific) and stored at − 20 °C until further analysis.

### Saturated oxytocin receptor autoradiography

Receptor autoradiography was performed for OTR using the [125I]-Ornithine Vasotocin Analog (d(CH2)5[Tyr(Me)2,Thr4,Orn8,[125I]Tyr9-NH2]-OVTA; (Perkin Elmer) as the hot ligand, while OT was used as the cold ligand to determine non-specific binding, as previously performed in our lab [37). The specificity of these ligands has been previously reported [38, 39].

Prior to beginning the experiment, the frozen slides were kept at room temperature for 1 h for acclimatization. Slides were then incubated in room temperature pre-incubation buffer (50 mM Tris-HCl, pH 7.4) twice for 5 min before being transferred into cold pre-incubation buffer. Next, the sections were placed in a humidified chamber surrounded by ice, and 800 µL of reaction mix containing50 pM [125I]-OVTA (specific activity:2200 Ci/mmol (PerkinElmer), 50 mM Tris-HCl (pH 7.4), 10 mM MgCl2, 0.1% bovine serum albumin, and 0.05% bacitracin was applied to each slide so that all sections were fully covered. Slides were incubated for 60 min at room temperature, and non-specific binding was determined by the addition of 2 µM OT (Tocris) into the incubation mix with [125I]-OVTA. Incubation was stopped by washing the sections three times with ice-cold washing buffer (50 mM Tris-HCl, 10 mM MgCl2) for 5 min, followed by dipping in ice-cold deionized water. Last, the sections were dried overnight under a stream of frigid air and left to dry in the cold room (4–6 °C).

To visualize and analyze the data, phosphor imaging plates (FUJI imaging plates, Storage Phosphor BAS-IP SR2025 Screen, GE Healthcare Life Sciences) were exposed for 72 h to the slides with brain sections and scanned in a phosphoimager (Fuji Phosphoimager Typhoon FLA 700, GE Healthcare Life Sciences), as previously described [38]. Digital images of the phosphor imaging-generated data were analyzed using MCID Image Analysis Software (InterFocus Imaging Ltd). Regions of interest (ROI) were defined based on anatomical landmarks, as illustrated in Fig. 7. The total and non-specific binding (in the presence of the cold ligand) was determined for each ROI on adjacent sections, and the non-specific signal was subtracted from the total signal of each ROI. As previously described, [125I]-quantitation standard curves (Amersham, GE Healthcare Life Sciences) were used to extrapolate the measured optical densities (photostimulable luminescence per mm2) of the tissue-equivalent OXTR densities from sections into nCi/mg [37). Binding in femtomoles per milligram (fmol/mg) was calculated according to the saturation binding equation (B = Bmax*[R]/(Kd +[R]), where Bmax represents the maximal bound receptor, Kd represents receptor affinity (Kd = 0.1 nM) in rat tissue [40], and [R] represents the concentration of the radioligand with which the specific activity of the radio ligand could be calculated. Data are defined as 0% (CTL) and changes in binding density show increase and decrease from baseline in Fig. 7 and raw data expressed as fmol/mg protein (mean ± SEM) can be found in the Supplementary Materials (Appendix 7 & 8).

### Data analysis

Since we collected data from two cohorts of rats (see Methods), we first tested for cohort differences with a student’s t-test. The cohorts did not differ statistically in any of the behavioral tests and therefore we proceeded with a combined analysis of both cohorts. The data analysis proceeded using univariate and mixed analysis of variance (ANOVAs). Statistically significant interactions, and main effects were followed up using Bonferroni-corrected pairwise comparisons, except when the interaction involved a within-group factor; paired t-tests were used. We report on all statistically significant main and interaction effects for clarity, only elaborating on the interaction effect. An alpha level of *p* < 0.05 (two-tailed) was set as the level of statistical significance, and we report partial eta squares as estimates of effect sizes or Hedge’s *g* along with individual data points for transparency. Statistical analyses were conducted using SPSS (29.0), and all graphs were illustrated in GraphPad Prism (10.0).

## Results

### Behavioral characterization of the persistent effect of adolescent social isolation

In short, we observed a general decrease in social recognition memory in both stress groups (EASI and LASI) regardless of sex. Both male and female LASI rats showed reduced social interactions. On the hotplate test, Male EASI and LASI rats demonstrated heightened thermal pain sensitivity, whereas the opposite was true for EASI females when compared to their sex-matched CTLs. We also observed some alterations to time spent in the center zone in male EASI rats and an increase total arm entries in female LASI rats.

### Elevated plus maze

**Fig. 3:**
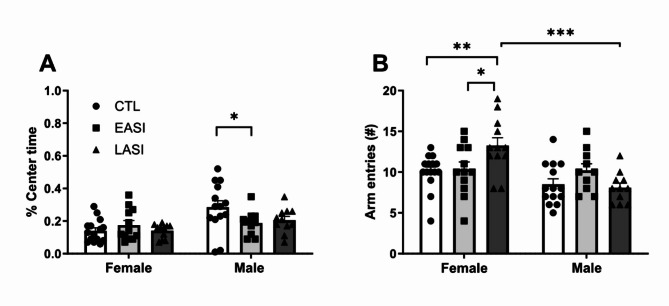
Behavioral performance in the elevated plus maze. (**A**) percent time spent in the center zone, (**B**) total arm entries in CTL (● control), early (■ EASI), and late (▲LASI) adolescent social isolation groups tested in adulthood (PD 90). Data are shown as individual data points with the mean ± SEM. *p < 0.5, **p < 0.01

The ANOVA performed on stress group (CTL, EASI, LASI) and sex (Male, Female) examined the effects of time spent on the open arm time, time spent in the center zone and total arm entries on anxiety-like and locomotor activity. The ANOVA on time spent on the open arm did not reveal a significant interaction effect (F _(1, 68)_ = 2.636, *p* = 0.079, ηp2 = 0.079) suggesting that ASI does not affect time spent on the open arm, regardless of sex. However, EASI males spent a lower percent of time in the center zone compared to CTL males (Fig. 3A), which may reflect an indirect measure of impulsive behavior [41]. Female LASI rats on the other hand showed higher general activity in the EPM compared to female CTL and EASI rats, as indicated by the total number of arm entries performed during the test.

The ANOVA for time spent in the center zone revealed an interaction between stress group and sex (F _(1, 68)_ = 3.199, *p* = 0.047, ηp2 = 0.086) and a main effect of sex (F _(1, 68)_ = 11.396, *p* = 0.001, ηp2 = 0.144). The stress group effect was not significant (F _(1, 68)_ = 1.324, *p* = 0.273, ηp2 = 0.037). To further explore the interaction effect, pairwise comparisons were conducted. Among males there was a significant difference in percent time spent on the center zone between male CTL (M = 0.285, SD = 0.149) and male EASI rats (M = 0.188 SD = 0.077) (Fig. 3A). The overall model was significant, (F_(1, 68)_ = 4.523, *p* = 0.001, ηp2 = 0.250), accounting for approximately 25% of the variance in time spent in the center zone.

The interaction effect between the stress group and sex for total number of arm entries was statistically significant (F _(1, 68)_ = 5.390 *p* = 0.0004, ηp2 = 0.165) as was the main effect of sex (F _(1, 68)_ = 13.395, *p* = 0.001, ηp2 = 0.144). Importantly, female LASI (M = 13.250, SD = 3.387) rats made more total entries compared to the other female groups; EASI (*p* = 0.009, M = 10.333, SD = 3.143), CTL (*p* = 0.027, M = 10.006, SD = 2.186) (Fig. 3B). Additionally, female LASI (M = 13.250, SD = 3.387) performed more total arm entries compared to male LASI (*p* < 0.00001, M = 8.090, SD = 1.868) rats (Fig. 3B). In general, female rats (M = 11.128, SD = 3.163) made more total arm entries than did male rats (*p* = 0.0004, M = 8.857, SD = 2.463). The overall model was significant, (F_(1, 68)_ = 5.643, *p* = 0.001, ηp2 = 0.293), accounting for approximately 29.3% of the variance of total arm entries.The different estrous cycle phases did not influence anxiety-like behavior (data not shown). Summary statistics in Supplementary materials.

### Open field

The ANOVA was conducted to investigate the effects of stress group (CTL, EASI, LASI) and sex (Male, Female) on total distance travelled on the OFT. We observed no long-lasting impact of ASI on the adult locomotor activity in the OFT (F _(1, 68)_ = 0.101 *p* = 0.904, ηp2 = 0.410) but observed a sex effect (F _(1, 68)_ = 51.395 *p* = 0.000001, ηp2 = 0.410). On average, female rats (M = 5653.987, SD = 1682.047) travelled a longer distance during the 30 min OFT compared to males (M = 3401.445, SD = 926.988) (Graph in Supplementary Materials). No effect was observed in the stress group (F _(1, 74)_ = 0.705, *p* = 0.498, ηp2 = 0.019). The overall model was significant, (F _(1, 68)_ = 10.990, *p* = 0.00001, ηp2 = 0.426), accounting for approximately 42.6% of the variance of total arm entries. Summary statistics in Supplementary materials.

### Novel object recognition memory

NOR data was analyzed using stress group (CTL, EASI, LASI) and sex (Male, Female) ANOVA. Adult ASI rats did not differ in object recognition ability (F _(1, 74)_ = 2.283, *p* = 0.109, ηp2 = 0.058). The ANOVA revealed no statistical differences between stress groups or sex on time spent investigating the two same objects during the acquisition phase (P1) (F _(1, 74)_ = 0.911, *p* = 0.407, ηp2 = 0.024). In the test phase (P2) we first assessed if all rats preferences differed from chance, which was statistically significant (t _(79)_ = 12.095, *p* = 0.000001; Hedge’s *g* = 11.115) implying all rats recognition ability differed from chance.

A non-significant interaction between stress group and sex was found on object discrimination ability during the test phase (P2) (F _(1, 74)_ = 2.283, *p* = 0.109, ηp2 = 0.058). The main effect of stress group (F _(1, 74)_ = 0.915, *p* = 0.405, ηp2 = 0.024) and sex (F _(1, 74)_ = 2.946, *p* = 0.090, ηp2 = 0.038) was statistically non-significant. The overall model was also not statistically significant, (F _(1, 74)_ = 1.695, *p* = 0.146, ηp2 = 0.426), indicating that the model as a whole did not explain a significant proportion of the variance of objection recognition ability. Summary statistics in Supplementary materials.

### Social interaction test

The ANOVA was conducted to analyze the effects of stress group (CTL, EASI, LASI) and sex (Male, Female) on total social interaction time, anogenital and non-anogenital sniffing in a free social interaction test performed in open field arena. We did not observe stress group differences between males and females (F _(1, 74)_ = 0.287, *p* = 0.752, ηp2 = 0.008). However, the analysis revealed a main effect of stress group, whereby LASI rats spent less time in social interactions bouts (based on social interactions initiated by the experimental rat) than CTL rats. This was largely driven by a reduction in interaction times across both sexes in both LASI groups.

**Fig. 4:**
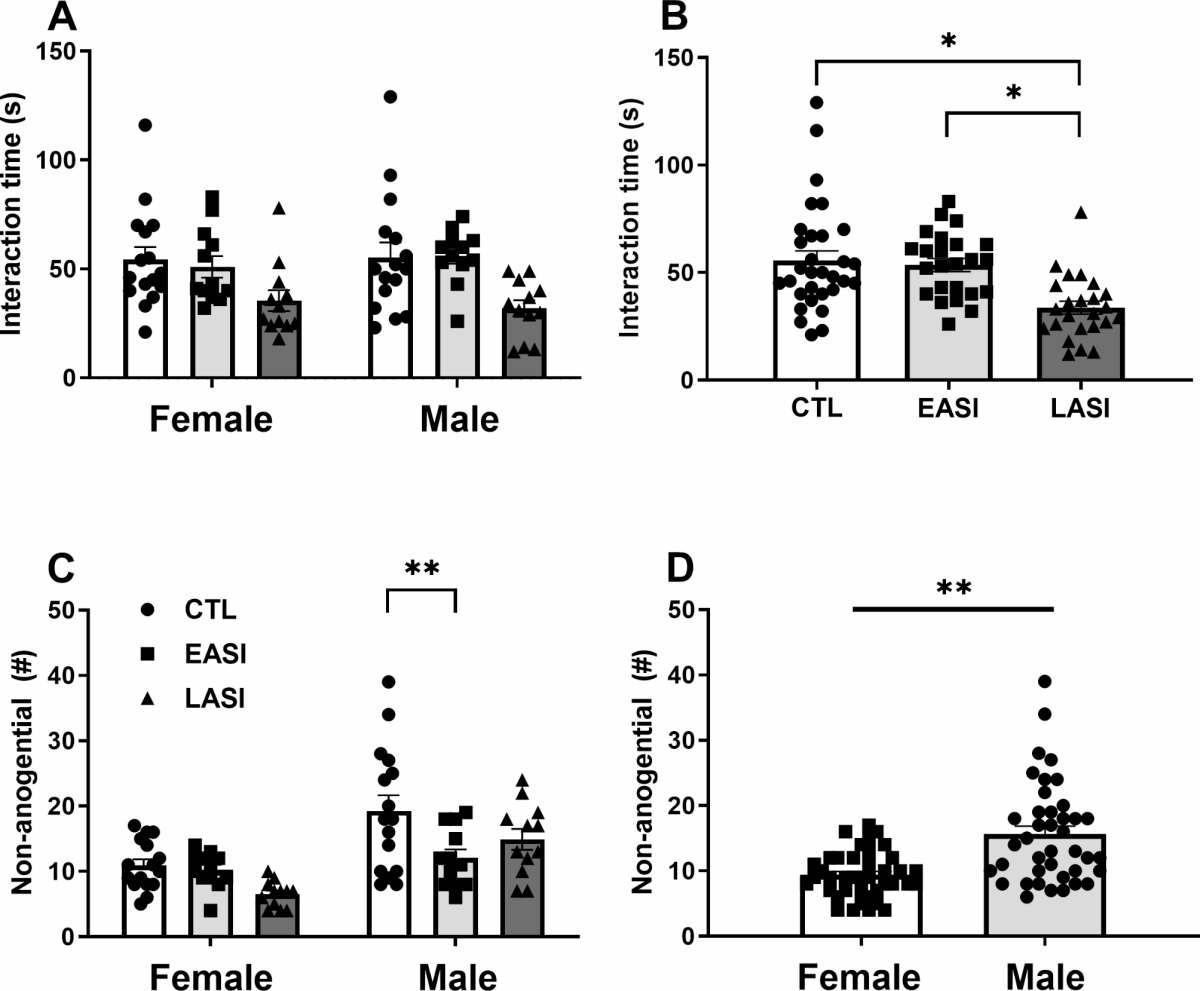
Behavioral performance in the social interaction test. (**A**) Total social interaction time in the social interaction test in the CTL (control), early (EASI), and late (LASI) adolescent social isolation groups tested in adulthood (PD 96) (SIT) across stress group and sex and (**B**) total interaction time by stress group (**C**) number of non-anogenital sniffing bouts and (**D**) sex difference in non-anogenital sniffing bouts between male and female rats. Data are shown as individual data points with the mean ± SEM. *p < 0.5, **p < 0.01

The main effect of stress group was statistically significant (F _(1, 74)_ = 9.036, *p* = 0.0003, η_p_2 = 0.196). Pairwise comparisons revealed LASI (M = 33.708, SD = 14.827) rats spent less time in social investigation than EASI (*p* = 0.003, M = 53.541, SD = 14.911) and CTL (*p* = 0.0005, M = 54.781, SD = 24.874) rats (Fig. 4B). The overall model was statistically significant (F _(1, 74)_ = 3.736, *p* = 0.005, ηp² = 0.202), suggesting that it accounts for 20.2% of the varianceof time spenting in social investigation during the social interaction test .

The ANOVA on non-anogenital sniffing indicated difference in bouts between the stress groups (F _(1, 74)_ = 5.483, *p* = 0.006, η_p_2 = 0.129) and sex (F _(1, 74)_ = 24.670, *p* = 0.000004, η_p_2 = 0.250) independently. Pair-wise comparisons between stress groups revealed that LASI (M = 10.708, SD = 5.901) rats performed fewer non-anogenital social investigation bouts than CTLs (*p* = 0.013, M = 15.093, SD = 8.294) and EASI (*p* = 0.031, M = 11.166, SD = 3.726) rats (Fig. 4C). While, male rats (M = 15.800, SD = 7.660) on average performed more non-anogential social bouts than female rats (*p* = 0.000004, M = 9.400, SD = 3.506) (Fig. 4D). The overall model was significant, (F _(1, 74)_ = 8.743, *p* = 0.000001, ηp2 = 0.371), accounting for approximately 37.1% of the variance of non-anogential sniffing bouts. We observed no effect of ASI on the frequency of anogenital sniffing bouts (F _(1, 74)_ = 3.001, *p* = 0.056, η_p_2 = 0.075). The overall model was not statistically significant (F _(1, 74)_ = 1.334, *p* = 0.260, ηp² = 0.083), suggesting that it did not account for a significant proportion of the variance of the anogential sniffing bouts.

The ANOVA revealed an interaction effect of the stress group and sex on rearing behaviors (F _(1, 74)_ = 4.341, *p* = 0.017, η_p_2 = 0.105) and a main effect of the stress group (F _(1, 74)_ = 8.887, *p* = 0.0003, η_p_2 = 0.194). Further analysis of the interaction effect revealed that male EASI (M = 17.666, SD = 5.804) rats reared less than LASI (*p* = 0.00001, M = 39.416), SD = 11.212) and CTL (*p* = 0.012, M = 29.687, SD = 16.684) rats, and male LASI rats reared more than LASI female rats (*p* = 0.008, M = 27.500, SD = 6.142). The overall model was statistically significant (F _(1, 74)_ = 5.939, *p* = 0.0001, η_p_2 = 0.286) accounting for approximately 28.6% of the variance of rearing in the SIT. Summary statistics in supplementary materials.

### Social recognition memory

The ANOVA was conducted on stress group (CTL, EASI, LASI) and sex (Male, Female) effects on investigation time (P1) and social recognition memory in the test phase (P2) based on investigations times between the novel and familiar conspecific. We found that in general ASI rats demonstrated impaired social recognition memory compared to CTL rats.

First, we assessed all rats’ preference against chance during the test phase (P2) before further analysis. To test this we performed a one sample t-test which was statistically significant (t _(79)_ = 45.569, *p* = 0.000001; Hedge’s *g* = 5.046), implying all rats differed from chance level in their discrimination ability.

**Fig. 5:**
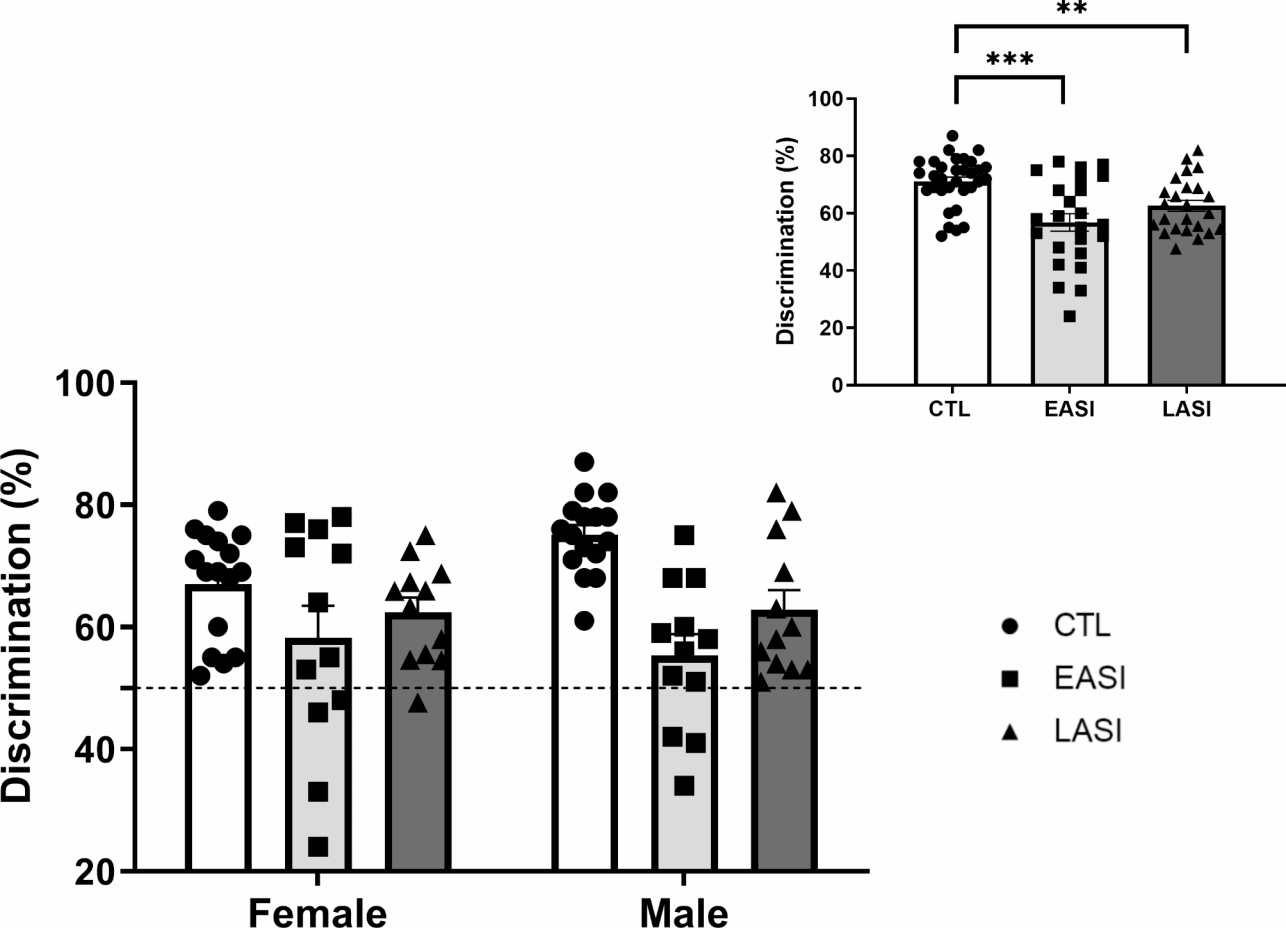
Behavioral performance on the social recognition memory test: The ability of rats to discriminate between social partners was indicated by a decrease in discrimination ability compared to CTL rats. The data are shown as individual data points with the mean ± SEM. *p < 0.5, **p < 0.01, ***p < 0.001. Main effect of stress group in small inlet (top right)

No differences were observed in the total time spent investigating both conspecifics during the test phase (P2) between stress groups independent of sex (F _(1, 74)_ = 0.219, *p* = 0.804, η_p_2 = 0.006). Neither of the main effect of stress group (F _(1, 74)_ = 0.794, *p* = 0.456, η_p_2 = 0.021) or sex (F _(1, 74)_ = 0.058, *p* = 0.810, η_p_2 = 0.021) were statistically significant. However, analysis of the social recognition memory discrimination ability revealed a main effect of stress group (F _(1, 74)_ = 11.241, *p* = 0.00003, η_p_2 = 0.241) (Fig. 5). Pairwise comparisons of the stress group variable revealed that EASI (*p* = 0.00002, M = 56.791, SD = 15.137) and LASI (*p* = 0.018, M = 62.632, SD = 9.524) rats showed impaired social recognition ability compared to CTL (M = 71.093, SD = 8.644) rats (Fig. 5, small inlet) regardless of sex. The overall model was statistically significant (F _(1, 74)_ = 0.794, *p* = 0.456, η_p_2 = 0.021) accounting for 27.65% of the variance in social recognition discrimination ability. Summary statistics in Supplementary materials.

#### Hotplate test

**Fig. 6:**
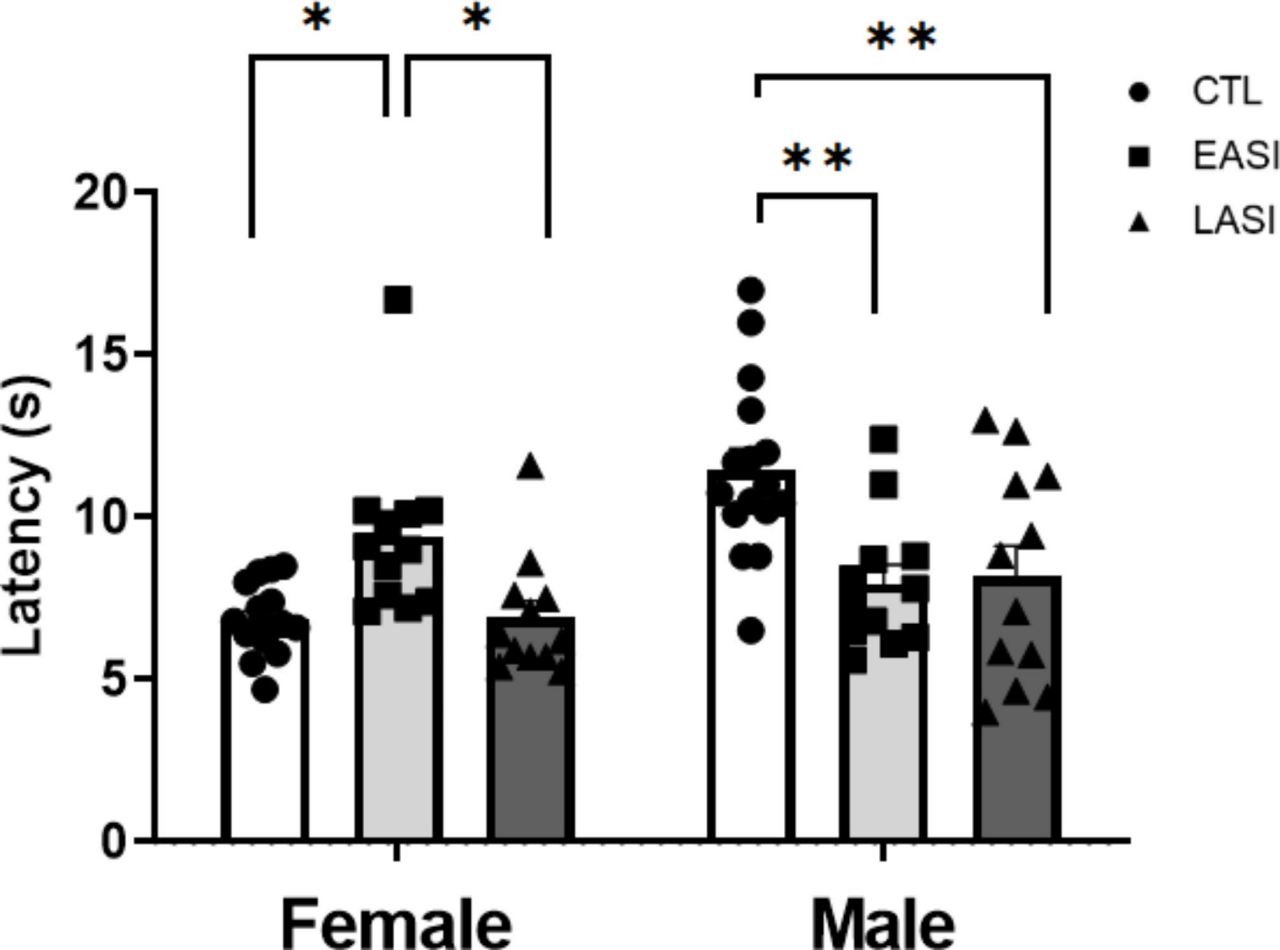
Behavioral performance on the hotplate test (52.5 °C). Latency to react to thermal pain stimuli in the hotplate test compared with controls (CTL). Data are shown as individual data points with the mean ± SEM. *p < 0.5, **p < 0.01, ***p < 0.001

The hotplate test data were analyzed using stress group (CTL, EASI, LASI) and sex (Male, Female) ANOVA. Compared to male CTL rats males EASI and LASI rats showed heightened thermal pain sensitivity. While female EASI rats in contrast demonstrated a reduction in thermal pain sensitivity in the hotplate test compared to female CTL rats. The interaction effects of stress group and sex (F _(1, 74)_ = 11.843, *p* = 0.00003, η_p_2 = 0.242) (Fig. 6) and the main effects of stress group (F _(1, 74)_ = 3.380, *p* = 0.039, η_p_2 = 0.084) and sex (F _(1, 74)_ = 7.812, *p* = 0.007, η_p_2 = 0.095) were statistically significant. Further exploration of the interaction effect revealed that both male EASI (*p* = 0.0005, M = 7.950, SD = 2.043) and LASI (*p* = 0.001, M = 7.950, SD = 2.043) rats demonstrated higher thermal pain sensitivity than male CTL rats. While, female EASI (M = 9.409, SD = 2.587) rats showed reduced thermal pain sensitivity compared to LASI (*p* = 0.031, M = 6.912, SD = 1.795) and CTL (*p* = 0.016, M = 6.862, SD = 1.074) rats. Male CTL (M = 9.418, SD = 3.134) rats demonstrated longer thermal latencies than female CTL (M = 7.641, SD = 2.148). We observed no differences in thermal pain sensitivity between estrous cycle phases captured immediately after the hotplate test. Summary statistics in Supplementary materials.

### Molecular characterization of the OTR binding in adulthood following adolescent social isolation

Data were analyzed using stress group (CTL, EASI, LASI) and sex (Male, Female) ANOVA to determine differences in OTR bindings levels in the several brain regions involved in social and anxiety-like behaviors.

**Fig. 7:**
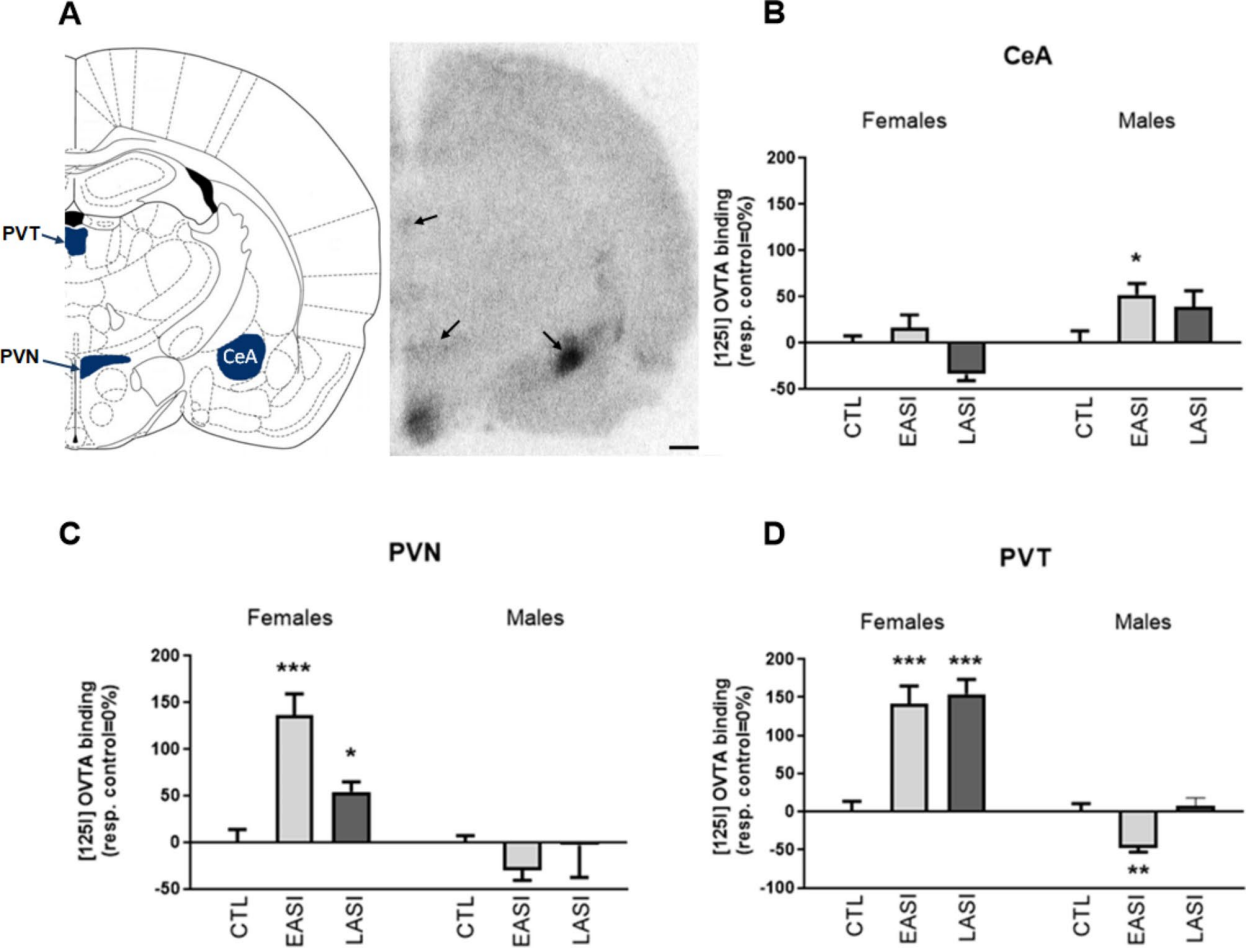
OTR binding sites in the CeA, PVN and PVT in adult rats (PD90) representative autoradiograph and bregma coordinates for regions of interest. (**A**) OTR bindings sites in the CeA, PVN, and PVT measured by saturated [125I] OVTA receptor autoradiography (fmol/mg). Bar graphs show OTR binding sites in the (**B**) CeA (mean female CTL values = 0.705 ± 0.05, mean male CTL values = 0.663 ± 0.08), (**C**) PVN (mean female CTL values = 0.124 ± 0.01, mean male CTL values = 0.205 ± 0.01), (**D**) PVT (mean female CTL values = 0.124 ± 0.01, mean male CTL values = 0.239 ± 0.02) are defined as 0% and changes in binding density show increase and decrease from baseline. Data shown as µ ± SEM. *p < 0.5, **p < 0.01, ***p < 0.001. Statistical analysis was performed by region-wise one-way ANOVA. *n* = 4–8/group. Scale bar 1 mm. CeA; central amygdala, PVN; paraventricular nucleus of the hypothalamus, PVT; paraventricular nucleus of the hypothalamus, CTL; control, EASI; early adolescent social isolation, LASI; late adolescent social isolation

We observed sex-dependent effects of ASI on OTR binding in the CeA, PVN, and PVT (Figs. 7; ; 8). In the PVT, OTR binding increased in female LASI and EASI by 154% and 141% respectively compared to female CTL rats. While in male EASI rats showed a 47% decrease in OTR binding compared male controls. While in the PVN OTR binding in female EASI and LASI rats increased by 136 and 54%, respectively. Male EASI rats showed a 52% increase in OTR binding in the CeA compared to CTL males.

**Fig. 8:**
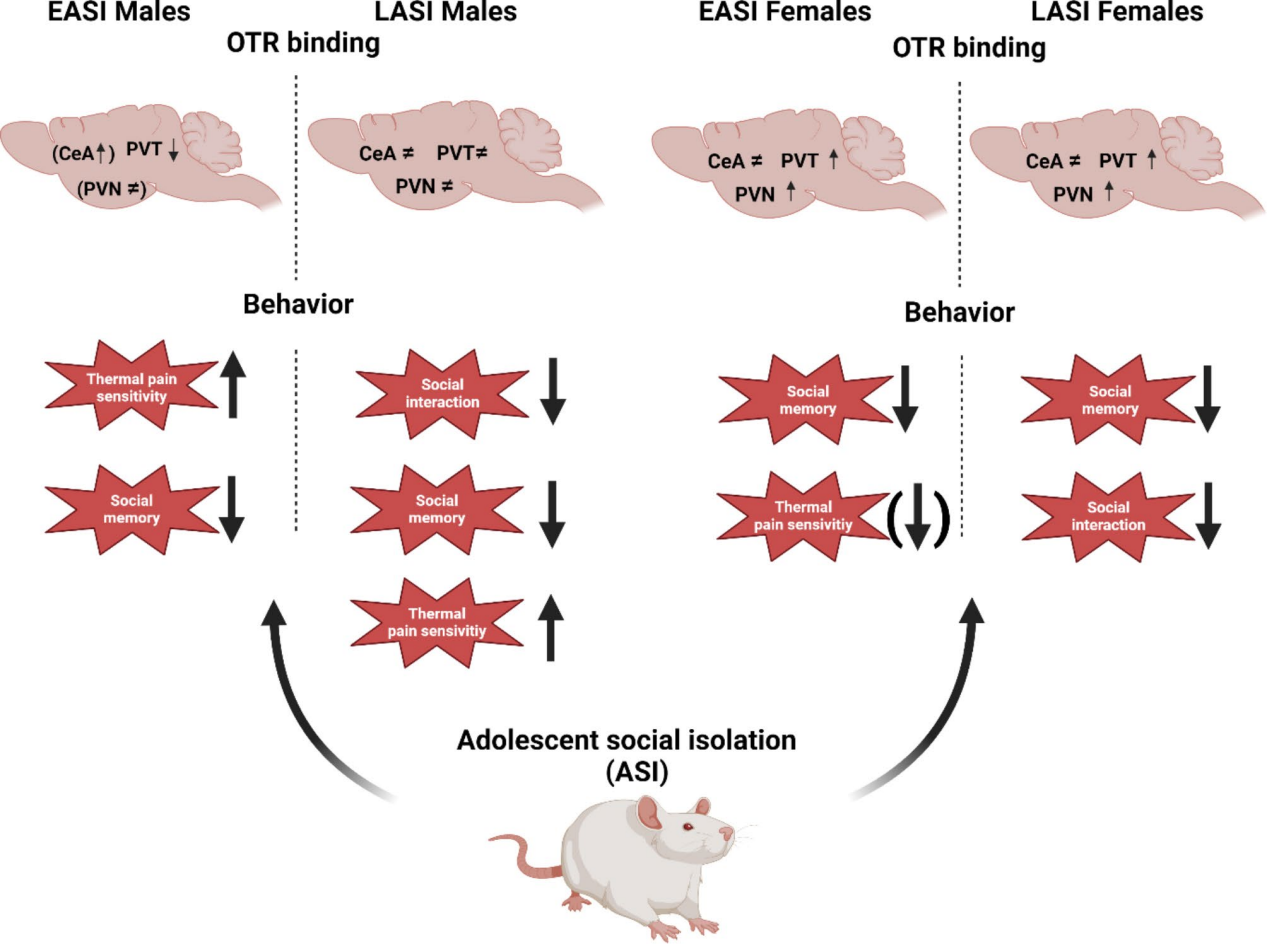
Main findings from the ASI study. Summary of behavioral and oxytocin receptor binding findings in early adolescent social isolation and late social isolation male and female rats compared to controls. Minor effects in brackets

For the PVT, we observed an interaction effect between stress group and sex (F = 150.791 _(1, 29)_, *p* = 0.00003, η_p_2 = 0.521), and the main effect of stress group (F = 70.870 _(1, 29)_, *p* = 0.002, η_p_2 = 0.352). Pairwise comparisons revealed that both EASI (*p* = 0.00005) and LASI (*p* = 0.00002) females had higher OTR binding levels in the PVT compared to CTL females. While male EASI rats demonstrated significantly less OTR binding levels compared to their CTL (*p* = 0.003) and LASI (*p* = 0.002) male counterparts. Interestingly, we observed an opposing effect of EASI on OTR binding in the PVT, with OTR binding increasing in females and but decreasing in males following EASI (*p* = 0.00001) (Appendix 8). Pair-wise comparisons of the sex and stress group interaction effects revealed that CTL females had lower levels of OTR binding compared to CTL males (*p* = 0.007) in adulthood.

In the PVN the interaction between stress group and sex (F = 80.267 _(1, 21)_, *p* = 0.002, η_p_2 = 0.441) was significant. Here, female EASI rats showed significantly more OTR binding in the PVN compared to female CTL (*p* = 0.0004) and LASI (*p* = 0.004) rats. We observed similar opposing sex specific effects in EASI rats in the PVN as we did in the PVT, with EASI leading to an increase in OTR binding in females but an opposing decrease in males (*p* = 0.001).

Analysis of the CeA revealed a significant stress group and sex interaction (F = 30.519 _(1, 21)_, *p* = 0.048, η_p_2 = 0.251), and the main effects of stress group (F = 30.904 _(1, 21)_, *p* = 0.036, η_p_2 = 0.271) and sex (F = 60.771 _(1, 21)_, *p* = 0.016, η_p_2 = 0.244). Pair-wise comparisons on the interaction effect demonstrated that female EASI had higher OTR binding levels compared to female LASI rats (*p* = 0.018) (Appendix 8). Additionally, OTR binding levels were higher in male EASI rats compared to male CTL rats (*p* = 0.016). We also observed sex differences between males LASI rats and female LASI (*p* = 0.002) rats were the former had significantly higher OTR binding levels. Post-hoc analysis for the stress group revealed statistically significant differences between the stress groups, with EASI rats demonstrating the highest OTR binding levels compared to CTL (*p* = 0.026) and LASI (*p* = 0.024). All statistical values are shown by sex for clarity but were analyzed together. Summary statistics in Supplementary Materials.

Figure 7 OTR binding sites in the CeA, PVN and PVT in adult rats (PD90) representative autoradiograph and bregma coordinates for regions of interest. (A) OTR bindings sites in the CeA, PVN, and PVT measured by saturated [125I] OVTA receptor autoradiography (fmol/mg). Bar graphs show OTR binding sites in the CeA (mean female CTL values = 0.705 ± 0.05, mean male CTL values = 0.663 ± 0.08), PVN (mean female CTL values = 0.124 ± 0.01, mean male CTL values = 0.205 ± 0.01), PVT (mean female CTL values = 0.124 ± 0.01, mean male CTL values = 0.239 ± 0.02) are defined as 0% and changes in binding density show increase and decrease from baseline. Data shown as µ ± SEM. *p < 0.5, **p < 0.01, ***p < 0.001. Statistical analysis was performed by region-wise one-way ANOVA. *n* = 4–8/group. Scale bar 1 mm. CeA; central amygdala, PVN; paraventricular nucleus of the hypothalamus, PVT; paraventricular nucleus of the hypothalamus, CTL; control, EASI; early adolescent social isolation, LASI; late adolescent social isolation.

#### Sex differences independent of adolescent social isolation

Additionally, we performed an analysis comparing male CTL rats to female CTL in order to identify sex difference independent of ASI. Across all tests, notable sex differences were observed in the EPM, OFT, SIT, and hotplate test, independent of ASI. The receptor autoradiography results revealed sex differences in the PVT between CTL males and females.

In the EPM male rats spent significantly more time in the central zone of the EPM compared to female CTL rats (F _(27)_ = 11.414, *p* = 0.002, η_p_2 = 0.297). On the other hand, female CTL rats made a significantly greater number of total arm entries (F _(27)_ = 26.773, *p* = 0.00001, η_p_2 = 0.472).

In the SIT, male rats engaged in more non-anogenital sniffing bouts compared to females (F _(30)_ = 10.496, *p* = 0.003, η_p_2 = 0.257), interestingly no significant sex differences were observed in the total time spent in social investigation (F _(30)_ = 0.011, *p* = 0.917, η_p_2 = 0.00). Additionally, no sex differences were noted in the SRM. In the hotplate test, males demonstrated a significantly higher thermal pain threshold on the hotplate test compared to females(F _(30)_ = 40.387, *p* = 0.000001, η_p_2 = 0.574).

The ANOVA on sex (male vs. female) conducted for the receptor autoradiography data revealed a general sex difference in OTR binding within the PVT (F _(29)_ = 30.421, *p* = 0.075, η_p_2 = 0.106), with males showing higher baseline levels of OTR binding compared to females (Appendix 8, Supplementary Materials).

## Discussion

In the present study, we characterized the long-term sequelae of EASI and LASI in male and female rats. To this end, we characterized the long-lasting impact of EASI and LASI across both sexes on social recognition memory and thermal pain sensitivity as well OTR binding in several key regions involved in social and anxiety-like behavior. We found that ASI regardless of timing of the initiation of ASI impairs social discrimination ability of both male and female rats in a domain specific manner (no alteration in object recognition) (Fig. 5). With LASI rats demonstrating deficits in the social interactions as well (Fig. 4). We also observed sex-dependent changes in thermal pain sensitivity (Fig. 6), with male EASI and LASI showing increased pain sensitivity compared to male CTL rats. While female EASI rats show a reduction to pain sensitivity compared to female CTL rats. On the EPM, EASI males spent less time in the center zone, suggesting these rats may be more impulsive than male control rats. Additionally, LASI females made more total arm entries (an indirect measure of locomotor activity) on the EPM compared to CTL females and LASI males. Finally, ASI induced long-lasting stress group and sex dependent alterations to OTR binding levels in the PVN and PVT and CeA (Fig. 7 provides an overview of all findings).

### LASI reduces social interactions in adulthood in both sexes

LASI rats of both sexes demonstrated a reduction in social interactions, which has been interpreted as increased social anxiety [42], but may also reflect reduced social interest or motivation [43]. Our findings did not agree with previous research in Wistar rats [16, 44]. Here, methodological differences could account for the observed discrepancies. For example, the study by Hol and colleagues (1999) investigated social behaviors across 20 min test sessions, while we used a 5 min test period. This effect on social interaction was not observed in EASI rats. An interpretation of the results is that LASI in both male and female rats is a more critical for the development of social behaviors than EASI. Thus, LASI covers a period between late adolescence and young adulthood a time when social interactions become more complex in nature and important for social development [45]. LASI may interfere with this development and lead to less sociability in adulthood. While the EASI group may have had time to recuperate from their ASI after resocialization on PD42 and hence, mitigated some of these effects. LASI also represents a period when rats still undergo a significant amount of neuronal pruning in brain regions known to modulate social behaviors such as the mPFC and AMY [46]. The authors in this study observed a significant reduction in dendritic spine between PD 42–56 but not PD 31–39 in the PFC. Hence, the absence of social contact during LASI may lead to different patterns of neural pruning in the mPFC and AMY, which could account for the reduction in social interactions in adulthood.

### ASI impairs social recognition memory in adulthood in both sexes

We found that both EASI and LASI male rats showed impairments in their social recognition memory, suggesting that ASI has general negative impact on social cognition. This effect appears to be domain specific, as we did not observe differences between CTL and ASI rats in the novel object recognition test. One consideration is that LASI rats spent less time in social interactions than CTL rats, which could explain why their social recognition memory was impaired. On the other hand, EASI rats did not demonstrate deficits in social interactions, yet displayed deficits in social recognition memory. It could suggest that there are two different mechanisms at play in altering social recognition memory between the EASI and LASI groups.

### ASI alters thermal pain sensitivity in a time- and sex-dependent manner

We observed pronounced sex and stress group differences in thermal pain sensitivity on the hotplate test (52.5 C). Both male EASI and LASI rats showed heightened sensitivity to thermal pain compared to control males. Conversely, female EASI rats exhibited a reduction in thermal pain sensitivity compared to both LASI and control females.

Our findings collectively underline the profound effect of ASI on social interactions, social recognition memory, and thermal pain sensitivity in adulthood and laid a foundation for follow up studies on identifying molecular targets. We chose the OT system as our target because of its crucial role in the social domain, where we found alterations in the CeA, PVN and PVT receptor binding.

## ASI alters the oxytocin receptor binding in several brain regions

OTR binding in the PVT exhibited a pronounced increase in female rats subjected to both EASI and LASI, while male EASI rats displayed a marked reduction in OTR binding. This divergence in OTR bindings suggests that the PVT’s response to ASI is highly timing and sex-dependent, potentially due to underlying neurobiological differences between males and females and their developmental trajectories [47].

In the PVN, a similar pattern emerged where female EASI rat’s demonstrated increased OTR binding compared to both CTL and LASI females, whereas male EASI rats showed a contrasting decrease compared to their male CTL counterparts. Furthermore, in the CeA, male EASI rats exhibited significantly higher OTR binding compared to male CTL rats, while female EASI rats had increased OTR binding relative to LASI females. The overall higher OTR binding in males across different stress conditions compared to females also suggests inherent sex differences in the oxytonergic system’s baseline functioning and ASI responsiveness.

The differential regulation of OTR binding in key brain regions associated with social and emotional behaviors indicates that males and females may employ distinct adaptive strategies in response to ASI, which may lead to changes in the OT system that induce the behavioral phenotypes observed. However, due to the multidirectionality of the OTR and behavioral findings it is difficult to interpret our findings together at this point in time. Hence, more studies on trying to dissect the molecular consequences of our OTR bindings on the observed behaviors still need to be conducted.

### Sex differences independent of ASI

We wanted to report on sex differences independent of ASI, which we think may be helpful feature for future researchers interested in sex differences within a “healthy” population of Wistar rats. Sex differences were apparent on the EPM, OFT, SIT, and hotplate test with CTL rats. Male CTL rats spent more time in the central zone than female CTLs on the EPM, as previously discussed; this may be an indirect indicator of impulsivity. Interestingly, we observed no sex differences in anxiety-like behavior on the EPM, a finding also previously reported in Wistar rats [48]. Most rat studies in different strains report female rats spending more time on the open arms, indicating a less anxious phenotype [42, 49–51]. These results suggest that strain differences may play a vital role in mediating sex differences in anxiety-like behavior on the EPM.

Next, female rats made more total number of arm entries, indicating females travelled a longer distance in the EPM than males. This finding is one of the most consistent sex differences found in the literature [42, 49, 51]. In the SIT male rats made more non-anogenital sniffing bouts than females (Fig. 4D), but did not differ in the time spent in social investigation. Nor did we observe sex differences on the SRM (Fig. 5).

Last, males demonstrated a higher thermal pain threshold compared to females on the hotplate test. Our finding aligns with the current literature on thermal pain in both rodents and humans. Where typically, women and female rats demonstrate a lower thermal pain threshold and higher pain sensitivity than males [52, 53]. In the receptor autoradiography data we observe a general sex difference in OTR binding in the CeA, male demonstrating higher leveling of OTR binding compared to females. In conclusion, we were able to replicate the most consistent sex differences (increased locomotor activity and higher thermal pain sensitivity in females) also observed in humans.

### Limitations

Some of the limitations that pertain to our study; Fatigue effects, which refer to rats becoming tired or less motivated in longer experiments. As we aimed to characterize the long-term effects this meant we had to rely on a large number of consecutive behavioral tests. Repeated testing can create fatigue effects. We tried our best to negate these with how we controlled the order of our experiments. We therefore tested rats every 48 h to allow them to recover between each test. Another consideration is a lack of direct ASI comparisons in the literature for most tests. Which highlights the need for more replication studies in the ASI field.

Next, the OTR alterations are difficult to interpret in conjunction with the observed behavioral changes due to the changes being so diverse. We think further functional studies with viral approaches to either up or down-regulate OTR in CeA, PVT and PVN, respectively are required to gain mechanistic insight.

#### Perspectives and significance

Although, our model cannot completely recapitulate human neuropsychiatric disorders or make direct comparisons, we can draw parallels from similarities in behavioral domains and neurobiological domains following ASI. For instance, we observed that the ASI model lead to persistent impairments in the social domain, in both social skills (social interactions) and cognition function (social recognition memory) as well as pain sensitivity (thermal). These changes are reminiscent of social problems, cognitive and chronic pain conditions in humans exposed to adolescent social isolation and other forms are adversity [54]. Taken together, our data suggest that the ASI paradigm has merit in the preclinical adversity field as a valid model that recapitulates key features of the human condition.

Klicken oder tippen Sie hier, um Text einzugeben.

## Electronic supplementary material

Below is the link to the electronic supplementary material.

Supplementary Material 1

## Data Availability

The manuscript has data included as electronic supplementary materials (summary statistics) and data will be made available on reasonable request.
