# Supplementary material for: Isolated during adolescence: long-term impact on social behavior, pain sensitivity, and the oxytocin system in male and female rats
Source: Biol Sex Differ. 2024 Oct 15;15:78. doi: 10.1186/s13293-024-00655-7 (PMC11476712; doi:10.1186/s13293-024-00655-7)
Supplement: Supplementary file 1 — Supplementary Material 1 [file 13293_2024_655_MOESM1_ESM.docx]

## **Adolescent social isolation supplementary materials**

**Appendix 1: Summary statistics for percent time spent on the open, closed arms, center zone and total number of arm entries on the elevated plus maze performed on PD 90**

| **Percent time spent on open arms** | | | | | | |
| --- | --- | --- | --- | --- | --- | --- |
| **Source** | **Type III Sum of Squares** | **df** | **Mean Square** | **F** | **Sig.** | **Partial Eta Squared** |
| **Corrected Model** | 0.218**^a^** | 5 | 0.044 | 2.428 | 0.044 | 0.151 |
| **Intercept** | 3.709 | 1 | 3.709 | 206.330 | <0.001 | 0.752 |
| **Stress group** | 0.105 | 2 | 0.053 | 2.924 | 0.061 | 0.079 |
| **Sex** | 0.023 | 1 | 0.023 | 1.280 | 0.262 | 0.018 |
| **Stress group * Sex** | 0.095 | 2 | 0.048 | 2.647 | 0.078 | 0.072 |
| **Error** | 1.222 | 68 | .018 |  |  |  |
| **Total** | 5.097 | 74 |  |  |  |  |
| **Corrected Total** | 1.441 | 73 |  |  |  |  |
| a. R Squared = 0.151 (Adjusted R Squared = 0.089) | | | | | | |

| **Percent time spent in center zone** | | | | | | |
| --- | --- | --- | --- | --- | --- | --- |
| **Source** | **Type III Sum of Squares** | **df** | **Mean Square** | **F** | **Sig.** | **Partial Eta Squared** |
| **Corrected Model** | 0.197**^a^** | 5 | 0.039 | 4.523 | 0.001 | 0.250 |
| **Intercept** | 2.599 | 1 | 2.599 | 297.967 | <0.001* | 0.814 |
| **Stress group** | 0.023 | 2 | 0.012 | 1.324 | 0.273 | 0.037 |
| **Sex** | **0.099** | **1** | **0.099** | **11.396** | **0.001** | **0.144** |
| **Stress group * Sex** | **0.056** | **2** | **0.028** | **3.199** | **0.047** | **0.086** |
| **Error** | 0.593 | 68 | 0.009 |  |  |  |
| **Total** | 3.450 | 74 |  |  |  |  |
| **Corrected Total** | 0.790 | 73 |  |  |  |  |
| a. R Squared = 0.250 (Adjusted R Squared = 0.194) | | | | | | |

| **Percent time spent in closed arms** | | | | | | |
| --- | --- | --- | --- | --- | --- | --- |
| **Source** | **Type III Sum of Squares** | **df** | **Mean Square** | **F** | **Sig.** | **Partial Eta Squared** |
| **Corrected Model** | 0.168^a^ | 5 | 0.034 | 1.267 | 0.288 | 0.085 |
| **Intercept** | 24.542 | 1 | 24.542 | 925.523 | <0.001 | 0.932 |
| **Stress group** | 0.052 | 2 | 0.026 | 0.989 | 0.377 | 0.028 |
| **Sex** | 0.021 | 1 | 0.021 | 0.774 | 0.382 | 0.011 |
| **Stress group * Sex** | .094 | 2 | 0.047 | 1.769 | 0.178 | 0.049 |
| **Error** | 1.803 | 68 | 0.027 |  |  |  |
| **Total** | 27.202 | 74 |  |  |  |  |
| **Corrected Total** | 1.971 | 73 |  |  |  |  |
| a. R Squared =.0,085 (Adjusted R Squared = 0.018) | | | | | | |

| **Total number of arm entries** | | | | | | |
| --- | --- | --- | --- | --- | --- | --- |
| **Source** | **Type III Sum of Squares** | **df** | **Mean Square** | **F** | **Sig.** | **Partial Eta Squared** |
| **Corrected Model** | 199.925^a^ | 5 | 39.985 | 5.643 | <0.001 | 0.293 |
| **Intercept** | 7370.017 | 1 | 7370.017 | 1040.057 | <0.001 | 0.939 |
| **Stress group** | 26.745 | 2 | 13.372 | 1.887 | 0.159 | 0.053 |
| **Sex** | **94.916** | **1** | **94.916** | **13.395** | **<0.001** | **0.165** |
| **Stress group * Sex** | **76.386** | **2** | **38.193** | **5.390** | **0.007** | **0.137** |
| **Error** | 481.859 | 68 | 7.086 |  |  |  |
| **Total** | 8162.000 | 74 |  |  |  |  |
| **Corrected Total** | 681.784 | 73 |  |  |  |  |
| a. R Squared = 0.293 (Adjusted R Squared = 0.241) | | | | | | |

**Appendix 2 Summary statistics and figure** **for distance travelled on the open field test on PD 92**

| **Total distance travelled in centimeters** | | | | | | |
| --- | --- | --- | --- | --- | --- | --- |
| **Source** | **Type III Sum of Squares** | **df** | **Mean Square** | **F** | **Sig.** | **Partial Eta Squared** |
| **Corrected Model** | 104544676.325^a^ | 5 | 20908935.265 | 10.990 | <0.001 | 0.426 |
| **Intercept** | 1599865722.946 | 1 | 1599865722.946 | 840.902 | <0.001 | 0.919 |
| **Stress group** | 2681346.934 | 2 | 1340673.467 | 0.705 | 0.498 | 0.019 |
| **Sex** | **98016872.555** | **1** | **98016872.555** | **51.518** | **<0.001** | **0.410** |
| **Stress group * Sex** | 384375.104 | 2 | 192187.552 | 0.101 | 0.904 | 0.003 |
| **Error** | 140789289.024 | 74 | 1902557.960 |  |  |  |
| **Total** | 1885351120.590 | 80 |  |  |  |  |
| **Corrected Total** | 245333965.349 | 79 |  |  |  |  |
| a. R Squared = 0.426 (Adjusted R Squared = 0.387) | | | | | | |

Adolescent social isolation did not influence adult performance on the open field test. Females rats travelled a longer distance during the 30 min OFT compared to males (F (1. 74) = 51.395 p < 0.000001. ηp2 = 0.410) in a sex-typical manner. Data shown with individual data points and µ±SEM.

**Appendix 3:** **Summary statistics, figure for rats’ discrimination ability as a percent of time spent investigating the novel vs. the familiar object during the test phase of the novel object recognition test performed on PD 94 and an image of the objects used for the test.**

| **Object discrimination ability** | | | | | | |
| --- | --- | --- | --- | --- | --- | --- |
| **Source** | **Type III Sum of Squares** | **df** | **Mean Square** | **F** | **Sig.** | **Partial Eta Squared** |
| **Corrected Model** | 984.050^a^ | 5 | 196.810 | 1.695 | 0.146 | 0.103 |
| **Intercept** | 328494.910 | 1 | 328494.910 | 2829.237 | <0.001 | 0.975 |
| **Stress group** | 212.435 | 2 | 106.218 | .915 | 0.405 | 0.024 |
| **Sex** | 342.046 | 1 | 342.046 | 2.946 | 0.090 | 0.038 |
| **Stress group * Sex** | 530.102 | 2 | 265.051 | 2.283 | 0.109 | 0.058 |
| **Error** | 8591.937 | 74 | 116.107 |  |  |  |
| **Total** | 346407.000 | 80 |  |  |  |  |
| **Corrected Total** | 9575.988 | 79 |  |  |  |  |
| a. R Squared = 0.103 (Adjusted R Squared = 0.042) | | | | | | |


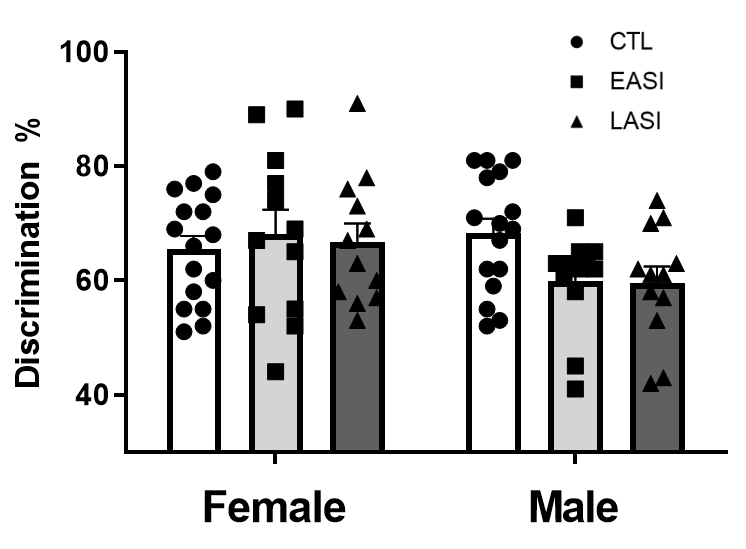


Adolescent social isolation did not influence adult novel object recognition (F _(1. 74)_ = 51.395 p < 0.000001. ηp2 = 0.410). Data shown with individual data points with µ±SEM.

**
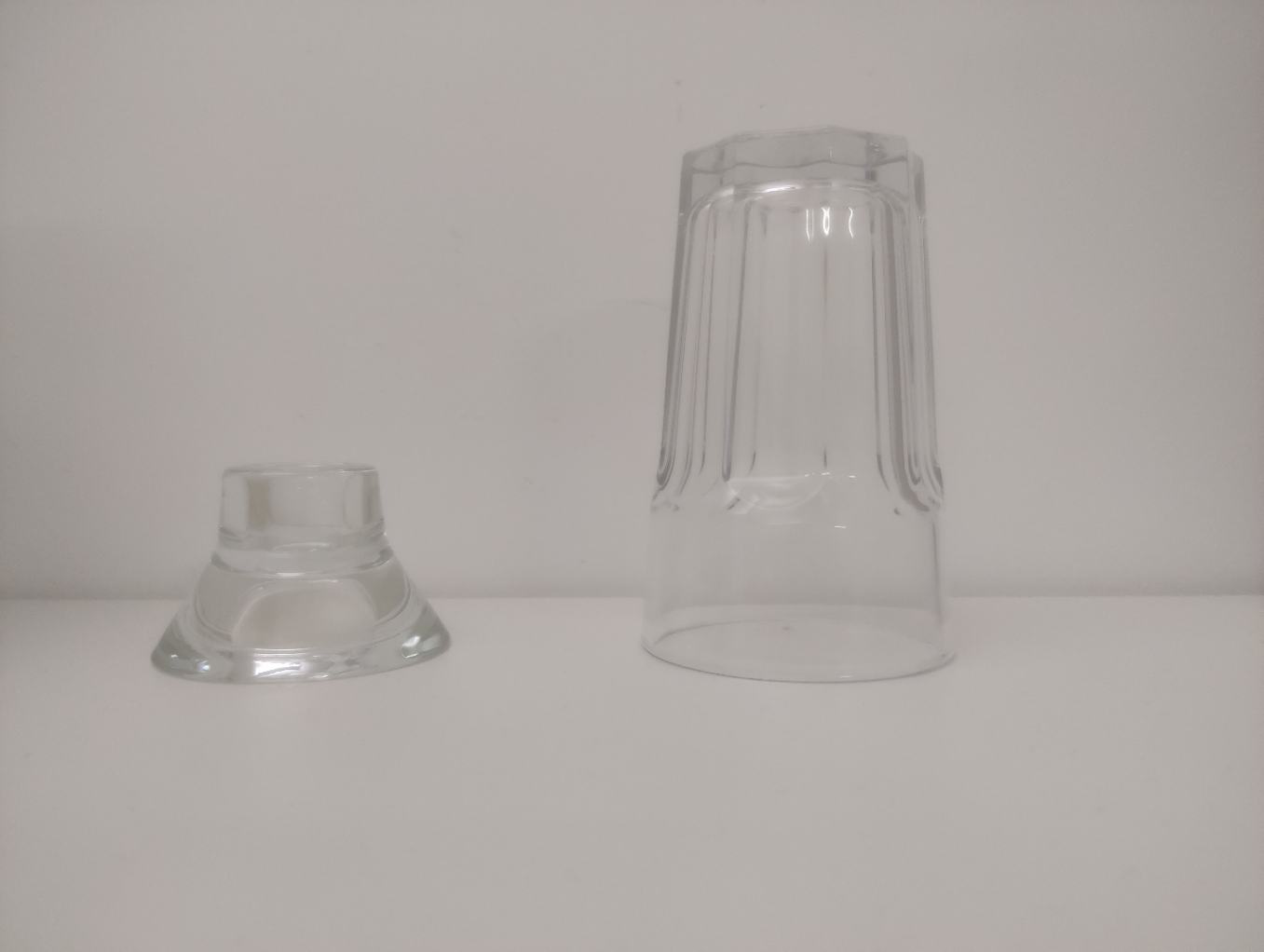
**

Both glassware was purchased from Ikea ([www.ikea.com](http://www.ikea.com))

**Appendix 4:** **Summary statistics for time spent in social interactions, frequency of anogenital sniffing, non-anogenital sniffing and rearing bouts in the social interaction test performed on PD 96**

| \| **Total time spent in social interactions** \| \| --- \| | | | | | | |
| --- | --- | --- | --- | --- | --- | --- | --- |
| **Source** | **Type III Sum of Squares** | **df** | **Mean Square** | **F** | **Sig.** | **Partial Eta Squared** |
| **Corrected Model** | 7349.117^a^ | 5 | 1469.823 | 3.736 | 0.005 | 0.202 |
| **Intercept** | 176054.190 | 1 | 176054.190 | 447.494 | <0.001 | 0.858 |
| **Stress group** | **7110.002** | **2** | **3555.001** | **9.036** | **<0.001** | **0.196** |
| **Sex** | 12.963 | 1 | 12.963 | 0.033 | 0.856 | 0.000 |
| **Stress group * Sex** | 225.502 | 2 | 112.751 | .287 | 0.752 | 0.008 |
| **Error** | 29113.271 | 74 | 393.423 |  |  |  |
| **Total** | 221455.000 | 80 |  |  |  |  |
| **Corrected Total** | 36462.387 | 79 |  |  |  |  |
| a. R Squared = 0.202 (Adjusted R Squared = 0.148) | | | | | | |

| **Anogenital sniffing bouts** | | | | | | |
| --- | --- | --- | --- | --- | --- | --- |
| **Source** | **Type III Sum of Squares** | **df** | **Mean Square** | **F** | **Sig.** | **Partial Eta Squared** |
| **Corrected Model** | 141.217^a^ | 5 | 28.243 | 1.334 | 0.260 | 0.083 |
| **Intercept** | 6465.690 | 1 | 6465.690 | 305.283 | <0.001 | 0.805 |
| **Stress group** | 127.102 | 2 | 63.551 | 3.001 | 0.056 | 0.075 |
| **Sex** | 9.281 | 1 | 9.281 | 0.438 | 0.510 | 0.006 |
| **Stress group * Sex** | 6.302 | 2 | 3.151 | 0.149 | 0.862 | 0.004 |
| **Error** | 1567.271 | 74 | 21.179 |  |  |  |
| **Total** | 8535.000 | 80 |  |  |  |  |
| **Corrected Total** | 1708.488 | 79 |  |  |  |  |
| a. R Squared = 0.083 (Adjusted R Squared = 0.021) | | | | | | |

| **Non-anogenital bouts** | | | | | | |
| --- | --- | --- | --- | --- | --- | --- |
| **Source** | **Type III Sum of Squares** | **df** | **Mean Square** | **F** | **Sig.** | **Partial Eta Squared** |
| **Corrected Model** | 1332.179^a^ | 5 | 266.436 | 8.743 | <0.001 | 0.371 |
| **Intercept** | 11927.463 | 1 | 11927.463 | 391.408 | <0.001 | 0.841 |
| **Stress group** | **334.190** | **2** | **167.095** | **5.483** | **0.006** | **0.129** |
| **Sex** | **751.781** | **1** | **751.781** | **24.670** | **<0.001** | **0.250** |
| **Stress group * Sex** | 178.790 | 2 | 89.395 | 2.934 | 0.059 | 0.073 |
| **Error** | 2255.021 | 74 | 30.473 |  |  |  |
| **Total** | 16288.000 | 80 |  |  |  |  |
| **Corrected Total** | 3587.200 | 79 |  |  |  |  |
| a. R Squared = 0.371 (Adjusted R Squared = 0.329) | | | | | | |

| **Rearing bouts** | | | | | | |
| --- | --- | --- | --- | --- | --- | --- |
| **Source** | **Type III Sum of Squares** | **df** | **Mean Square** | **F** | **Sig.** | **Partial Eta Squared** |
| **Corrected Model** | 3348.800^a^ | 5 | 669.760 | 5.939 | <0.001 | .0.286 |
| **Intercept** | 56774.668 | 1 | 56774.668 | 503.473 | <0.001 | 0.872 |
| **Stress group** | **2004.352** | **2** | **1002.176** | **8.887** | **<0.001** | **0.194** |
| **Sex** | 326.296 | 1 | 326.296 | 2.894 | 0.093 | 0.038 |
| **Stress group * Sex** | **978.935** | **2** | **489.468** | **4.341** | **0.017** | **0.105** |
| **Error** | 8344.688 | 74 | 112.766 |  |  |  |
| **Total** | 69421.000 | 80 |  |  |  |  |
| **Corrected Total** | 11693.488 | 79 |  |  |  |  |
| a. R Squared = 0.286 (Adjusted R Squared = 0.238) | | | | | | |

**Appendix 5: Summary statistics for duration spent in investigating the novel, familiar, both conspecifics and social discrimination ability in the social recognition memory test performed on PD 96**

| **Duration spent investigating the novel conspecific** | | | | | | |
| --- | --- | --- | --- | --- | --- | --- |
| **Source** | **Type III Sum of Squares** | **df** | **Mean Square** | **F** | **Sig.** | **Partial Eta Squared** |
| **Corrected Model** | 228.029^a^ | 5 | 45.606 | .314 | 0.903 | 0.021 |
| **Intercept** | 39176.727 | 1 | 39176.727 | 269.984 | <0.001 | 0.785 |
| **Stress group** | 104.363 | 2 | 52.181 | 0.360 | 0.699 | 0.010 |
| **Sex** | 34.186 | 1 | 34.186 | 0.236 | 0.629 | 0.003 |
| **Stress group * Sex** | 74.054 | 2 | 37.027 | 0.255 | 0.775 | 0.007 |
| **Error** | 10737.958 | 74 | 145.108 |  |  |  |
| **Total** | 51421.000 | 80 |  |  |  |  |
| **Corrected Total** | 10965.988 | 79 |  |  |  |  |
| a. R Squared = 0.021 (Adjusted R Squared = 0.045) | | | | | | |

| **Duration spent investigating familiar conspecific** | | | | | | |
| --- | --- | --- | --- | --- | --- | --- |
| **Source** | **Type III Sum of Squares** | **df** | **Mean Square** | **F** | **Sig.** | **Partial Eta Squared** |
| **Corrected Model** | 1163.533^a^ | 5 | 232.707 | 3.360 | 0.009 | 0.185 |
| **Intercept** | 14131.410 | 1 | 14131.410 | 204.030 | <0.001 | 0.734 |
| **Stress group** | **930.085** | **2** | **465.043** | **6.714** | **0.002** | **0.154** |
| **Sex** | 2.660 | 1 | 2.660 | 0.038 | 0.845 | 0.001 |
| **Stress group * Sex** | 222.935 | 2 | 111.468 | 1.609 | 0.207 | 0.042 |
| **Error** | 5125.354 | 74 | 69.262 |  |  |  |
| **Total** | 19887.000 | 80 |  |  |  |  |
| **Corrected Total** | 6288.888 | 79 |  |  |  |  |
| a. R Squared = 0.185 (Adjusted R Squared = 0.130) | | | | | | |

| **Total interaction duration for both conspecifics** | | | | | | |
| --- | --- | --- | --- | --- | --- | --- |
| **Source** | **Type III Sum of Squares** | **df** | **Mean Square** | **F** | **Sig.** | **Partial Eta Squared** |
| **Corrected Model** | 636.596^a^ | 5 | 127.319 | 0.415 | 0.837 | 0.027 |
| **Intercept** | 100366.501 | 1 | 100366.501 | 327.166 | <0.001 | 0.816 |
| **Stress group** | 487.523 | 2 | 243.761 | 0.795 | 0.456 | 0.021 |
| **Sex** | 17.774 | 1 | 17.774 | 0.058 | 0.810 | 0.001 |
| **Stress group * Sex** | 134.623 | 2 | 67.311 | 0.219 | 0.804 | 0.006 |
| **Error** | 22701.354 | 74 | 306.775 |  |  |  |
| **Total** | 124300.000 | 80 |  |  |  |  |
| **Corrected Total** | 23337.950 | 79 |  |  |  |  |
| a. R Squared = 0.027 (Adjusted R Squared = 0.038) | | | | | | |

| **Social discrimination ability** | | | | | | |
| --- | --- | --- | --- | --- | --- | --- |
| **Source** | **Type III Sum of Squares** | **df** | **Mean Square** | **F** | **Sig.** | **Partial Eta Squared** |
| **Corrected Model** | 3468.638^a^ | 5 | 693.728 | 5.641 | <0.001 | 0.276 |
| **Intercept** | 316774.486 | 1 | 316774.486 | 2575.626 | <0.001 | 0.972 |
| **Stress group** | **2896.597** | **2** | **1448.298** | **11.776** | **<0.001** | **0.241** |
| **Sex** | 67.145 | 1 | 67.145 | .546 | 0.462 | 0.007 |
| **Stress group * Sex** | 449.974 | 2 | 224.987 | 1.829 | 0.168 | 0.047 |
| **Error** | 9101.210 | 74 | 122.989 |  |  |  |
| **Total** | 342966.495 | 80 |  |  |  |  |
| **Corrected Total** | 12569.848 | 79 |  |  |  |  |
| a. R Squared = 0.276 (Adjusted R Squared = 0.227) | | | | | | |

**Appendix 6: Summary statistics for latency (s) to a thermal pain stimulus (52.5°C) on the hotplate test performed on PD 98 in study 1A**

| **Latency to a thermal pain stimulus** | | | | | | |
| --- | --- | --- | --- | --- | --- | --- |
| **Source** | **Type III Sum of Squares** | **df** | **Mean Square** | **F** | **Sig.** | **Partial Eta Squared** |
| **Corrected Model** | 227.299^a^ | 5 | 45.460 | 8.432 | <0.001 | 0.363 |
| **Intercept** | 5622.039 | 1 | 5622.039 | 1042.781 | <0.001 | 0.934 |
| **Stress group** | **36.449** | **2** | **18.225** | **3.380** | **0.039** | **0.084** |
| **Sex** | **42.116** | **1** | **42.116** | **7.812** | **0.007** | **0.095** |
| **Stress group * Sex** | **127.695** | **2** | **63.848** | **11.843** | **<0.001** | **0.242** |
| **Error** | 398.963 | 74 | 5.391 |  |  |  |
| **Total** | 6447.134 | 80 |  |  |  |  |
| **Corrected Total** | 626.262 | 79 |  |  |  |  |
| a. R Squared = ,363 (Adjusted R Squared = ,320) | | | | | | |

**Appendix 7: Summary statistic for [^125^I]-OVTA receptor binding sites (fmol/mg) from male and female rats separately**

| **Males** | | | | | | |
| --- | --- | --- | --- | --- | --- | --- |
| **Binding sites** | **Region** | **CTL ♂** | **EASI ♂** | **LASI ♂** | **F-value** | **Interaction**  **P-value** |
| ([^125^I]-OVTA | DLS | 0.619±0.4  N = 8 | 0.546±0.05  N = 8 | 0.608±0.02  N= 8 | 1.301 (1. 37) | 0.284 |
|  | DMS | 0.678±0.01  N = 8 | 0.632±0.03  N = 8 | 0.655±0.02  N = 8 | 1.430 (1. 36) | 0.237 |
|  | CPU | 0.291±0.03  N = 7 | 0.273±0.04  N = 8 | 0.250±0.05  N = 6 | 0.571 (1. 28) | 0.721 |
|  | ACB | 0.291±0.03  N = 8 | 0.210±0.02  N = 7 | 0.26±0.07  N = 4 | 0.817 (1. 30) | 0.547 |
|  | **PVN** | **0.205±0.01**  N = 5 | **0.143±0.02**  N = 3 | **0.199±0.07**  N = 3 | **80.267 (1. 21)** | **0.002** |
|  | **CEA** | **0.663****±0.08**  N = 4 | **1.008±0.08**  N = 5 | **0.919±0.11**  N = 6 | **30.519 (1. 21)** | **0.048** |
|  | BLA | 0.121±0.00  N =4 | 0.193±0.05  N = 5 | 0.164±0.02  N = 6 | 2.018 (1. 19) | 0.1220. |
|  | **PVT** | **0.239±0.02**  N = 6 | **0125±0.01**  N = 6 | **0.257±0.02**  N = 4 | **150.791 (1. 29)** | **0.00003** |
|  | vCA1 | 0.869±0.08  N = 5 | 1.173±0.13  N = 6 | 1.070±0.04  N = 4 | 1.520 (1. 25) | 0.219 |

| **Females** | | | | | | |
| --- | --- | --- | --- | --- | --- | --- |
| **Binding sites** | **Region** | **CTL** ♁ | **EASI** ♁ | **LASI** ♁ | **F-value** | **P-value** |
| ([^125^I]-OVTA | DLS | 0.614±0.3  N = 6 | 0.628±0.02  N = 7 | 0.688±0.03  N = 6 | 1.301 (1. 37) | 0.284 |
|  | DMS | 0.696±0.02  N = 5 | 0.643±0.02  N = 8 | 0.711±0.02  N = 6 | 1.430 (1. 36) | 0.237 |
|  | CPU | 0.359±0.04  N = 4 | 0.261±0.04  N = 4 | 0.295±0.03  N = 5 | 0.571 (1. 28) | 0.721 |
|  | ACB | 0.308±0.04  N = 4 | 0.234±0.06  N = 6 | 0.284±0.03  N = 6 | 0.817 (1. 30) | 0.547 |
|  | **PVN** | **0.124±0.01**  N = 3 | **0.295±0.02**  N = 6 | **0.192±0.01**  N = 7 | **80.267 (1. 21)** | **0.002** |
|  | **CEA** | **0.705±0.05**  N =4 | **0.822±0.09**  N = 4 | **0.465±0.04**  N =4 | **30.519 (1. 21)** | **0.048** |
|  | BLA | 0.182±0.04  N = 4 | 0.179±0.00  N = 4 | 0.089±0.00  N = 4 | 2.018 (1. 19) | 0.1220. |
|  | **PVT** | **0.124±0.01**  N = 4 | **0.299±0.02**  N = 8 | **0.316±0.02** N = 7 | **150.791 (1. 29)** | **0.00003** |
|  | vCA1 | 1.072±0.07  N = 6 | 1.032±0.09  N = 5 | 1.205±0.09  N = 5 | 1.520 (1. 25) | 0.219 |

**Table 2: [^125^I]-OVTA receptor autoradiography in ASI male and female rats. All receptor/transporter autoradiography was performed under saturated conditions. Kd-values, the dissociation equilibrium constant describing the affinity for a specific receptor, as well as Bmax-values, describing the maximum density of the receptor in the specified regions. DLS. dorsal-lateral striatum; DMS. dorsal-medial striatum; CPU. caudate putamen; ACB. nucleus accumbens core; PVN. paraventricular nucleus of the hypothalamus.; CeA. central amygdala; BLA. basolateral amygdala; PVT. paraventricular nucleus of the thalamus; vCA1. ventral cornu Ammonis 1. Statistics performed across all groups and both sexes. All values shown in fmol/g with Mean±SEM.**

**Appendix 8: Summary statistics and bar graphs for sex comparisons for OT receptor autoradiography (fmol/p/g) for PVT, PVN and CeA**

Bar graph shows sex comparisons between groups in OTR bindings sites (fmol/mg) in the PVT measured by saturated [125I] OVTA receptor autoradiography. Data shown with individual data points, µ±SEM. *****p < 0.5. Summary statistics, and pair-wise comparisons for the PVT are shown below.

| **Tests of Between-Subjects Effects** | | | | | | |
| --- | --- | --- | --- | --- | --- | --- |
| **Dependent Variable: PVT** | | | | | | |
| **Source** | **Type III Sum of Squares** | **df** | **Mean Square** | **F** | **Sig.** | **Partial Eta Squared** |
| **Corrected Model** | 0.202a | 5 | 0.040 | 100.842 | <0.001 | 0.651 |
| **Intercept** | 10.684 | 1 | 10.684 | 4510.852 | <0.001 | 0.940 |
| Sex | 0.013 | 1 | 0.013 | 30.421 | 0.075 | 0.106 |
| **Stress group** | **0.059** | **2** | **0.029** | **70.871** | **0.002** | **0.352** |
| **Sex * Stress group** | **0.118** | **2** | **0.059** | **150.791** | **<0.001** | **0.521** |
| **Error** | 0.108 | 29 | 0.004 |  |  |  |
| **Total** | 20.290 | 35 |  |  |  |  |
| **Corrected Total** | 0.310 | 34 |  |  |  |  |
| a. R Squared = 0.651 (Adjusted R Squared = 0.591) | | | | | | |

| **Pairwise Comparisons: Sex * Stress group** | | | | | | | | | | | |
| --- | --- | --- | --- | --- | --- | --- | --- | --- | --- | --- | --- |
| **Dependent Variable: PVT** | | | | | | | | | | | |
| **Stress group** | **(I) Sex** | | **(J) Sex** | | | **Mean Difference (I-J)** | **Std. Error** | **Sig.** | | **95% Confidence Interval** | |
|  |  |  |  |  |  |  |  |  |  | **LB** | **UB** |
| **CTL** | | **female** | | **male** | **-0.115*** | | **0.039** | **0.007** | **-0.195** | | **-0.034** |
| **EASI** | | **female** | | **male** | **0.174*** | | **0.033** | **<0.001** | **0.107** | | **0.242** |
| LASI | | female | | male | 0.059 | | 0.038 | 0.134 | -0.019 | | 0.137 |
| Based on estimated marginal means | | | | | | | | | | | |
| *. The mean difference is significant at the 0.05 level. | | | | | | | | | | | |

| **Pairwise Comparisons: Stress * Sex** | | | | | | | |
| --- | --- | --- | --- | --- | --- | --- | --- |
| **Dependent Variable: PVT** | | | | | | | |
| **Sex** | **(I) Stress group** | **(J) Stress group** | **Mean Difference (I-J)** | **Std. Error** | **Sig.** | **95% Confidence Interval** | |
|  |  |  |  |  |  | **LB** | **UB** |
| **female** | **CTL** | **EASI** | **-0.175*** | **0.037** | **<0.001** | **-0.252** | **-0.099** |
|  |  | LASI | **-0.192*** | **0.038** | **<0.001** | **-0.270** | **-0.113** |
|  | EASI | CTL | 0.175* | 0.037 | <0.001 | 0.099 | 0.252 |
|  |  | LASI | -0.016 | 0.032 | 0.609 | -0.081 | 0.048 |
|  | LASI | CTL | 0.192* | 0.038 | <0.001 | 0.113 | 0.270 |
|  |  | EASI | 0.016 | 0.032 | 0.609 | -0.048 | 0.081 |
| **male** | **CTL** | **EASI** | **0.113^*^** | **0.035** | **0.003** | **0.041** | **0.185** |
|  |  | LASI | -0.018 | 0.039 | 0.649 | -0.099 | 0.062 |
|  | EASI | CTL | -0.113^*^ | 0.035 | 0.003 | -0.185 | -0.041 |
|  |  | LASI | -0.131^*^ | 0.039 | 0.002 | -0.212 | -0.051 |
|  | **LASI** | CTL | 0.018 | 0.039 | 0.649 | -0.062 | 0.099 |
|  |  | **EASI** | **0.131^*^** | **0.039** | **0.002** | **0.051** | **0.212** |
| Based on estimated marginal means | | | | | | | |
| *. The mean difference is significant at the 0.05 level. | | | | | | | |

Bar graph shows sex comparisons between groups in OTR bindings sites (fmol/mg) in the PVN measured by saturated [125I] OVTA receptor autoradiography. Data shown with individual data points, µ±SEM. *****p < 0.5. Summary statistics, and pair-wise comparisons for the PVN are shown below.

| **Tests of Between-Subjects Effects** | | | | | | |  |
| --- | --- | --- | --- | --- | --- | --- | --- |
| **Dependent Variable: PVN** | | | | | | |  |
| **Source** | **Type III Sum of Squares** | **df** | **Mean Square** | **F** | **Sig.** | **Partial Eta Squared** |  |
| **Corrected Model** | 0.081^a^ | 5 | 0.016 | 40.835 | 0.004 | 0.535 |  |
| **Intercept** | 0.892 | 1 | 0.892 | 2660.926 | <0.001 | 0.927 |  |
| **Sex** | 0.003 | 1 | 0.003 | 0.821 | 0.375 | 0.038 |  |
| **Stress group** | 0.011 | 2 | 0.006 | 10.703 | 0.206 | 0.140 |  |
| **Sex * Stress group** | **0.055** | **2** | **0.028** | **80.267** | **0.002** | **0.441** |  |
| **Error** | 0.070 | 21 | 0.003 |  |  |  |  |
| **Total** | 10.291 | 27 |  |  |  |  |  |
| Corrected Total | 0.151 | 26 |  |  |  |  |  |
| a. R Squared = 0.535 (Adjusted R Squared = 0.424) | | | | | | |  |

| **Pairwise Comparisons: Sex * Stress group** | | | | | | | | | | |
| --- | --- | --- | --- | --- | --- | --- | --- | --- | --- | --- |
| **Dependent Variable: PVN** | | | | | | | | | | |
| **Stress group** | **(I) Sex** | **(J) Sex** | | **Mean Difference (I-J)** | **Std. Error** | **Sig.** | | **95% Confidence Interval** | | |
|  |  |  |  |  |  |  |  | **LB** | **UB** | |
| CTL | female | male | -0.081 | | 0.042 | 0.069 | -0.169 | | | 0.007 |
| **EASI** | **female** | **male** | **0.152*** | | **0.041** | **0.001** | **0.067** | | | **0.237** |
| LASI | female | male | -0.007 | | 0.040 | 0.869 | -0.090 | | | 0.076 |
| Based on estimated marginal means | | | | | | | | | | |
| *. The mean difference is significant at the 0.05 level. | | | | | | | | | | |

| **Pairwise Comparisons: Stress * Sex** | | | | | | | |
| --- | --- | --- | --- | --- | --- | --- | --- |
| **Dependent Variable: PVN** | | | | | | | |
| **Sex** | **(I) Stress group** | **(J) Stress group** | **Mean Difference (I-J)** | **Std. Error** | **Sig.** | **95% Confidence Interval** | |
|  |  |  |  |  |  | **Lower Bound** | **Upper Bound** |
| **female** | **CTL** | **EASI** | **-0.170*** | **0.041** | **<0.001** | **-0.255** | **-0.085** |
|  |  | LASI | -0.068 | 0.040 | 0.105 | -0.151 | 0.015 |
|  | **EASI** | CTL | 0.170* | 0.041 | <0.001 | 0.085 | 0.255 |
|  |  | **LASI** | **0.103*** | **0.032** | **0.004** | **0.036** | **0.170** |
|  | LASI | CTL | 0.068 | 0.040 | 0.105 | -0.015 | 0.151 |
|  |  | EASI | -0.103* | 0.032 | 0.004 | -0.170 | -0.036 |
| **male** | **CTL** | **EASI** | 0.062 | 0.042 | 0.156 | -0.026 | 0.150 |
|  |  | LASI | 0.007 | 0.042 | 0.877 | -0.081 | 0.094 |
|  | EASI | CTL | -0.062 | 0.042 | 0.156 | -0.150 | 0.026 |
|  |  | LASI | -0.056 | 0.047 | 0.252 | -0.154 | 0.043 |
|  | **LASI** | CTL | -0.007 | 0.042 | 0.877 | -0.094 | 0.081 |
|  |  | **EASI** | 0.056 | 0.047 | 0.252 | -0.043 | 0.154 |
| Based on estimated marginal means | | | | | | | |
| *. The mean difference is significant at the 0.05 level. | | | | | | | |

Bar graph shows sex comparisons between groups in OTR bindings sites (fmol/mg) in the CeA measured by saturated [125I] OVTA receptor autoradiography. Data shown with individual data points, µ±SEM. Summary statistics, and pair-wise comparisons for the CeA are shown below.

| **Tests of Between-Subjects Effects** | | | | | | |
| --- | --- | --- | --- | --- | --- | --- |
| **Dependent Variable: CeA** | | | | | | |
| **Source** | **Type III Sum of Squares** | **df** | **Mean Square** | **F** | **Sig.** | **Partial Eta Squared** |
| **Corrected Model** | 0.856a | 5 | 0.171 | 40.429 | 0.007 | 0.513 |
| **Intercept** | 150.377 | 1 | 150.377 | 3970.592 | <0.001 | 0.950 |
| **Stress group** | **0.302** | **2** | **0.151** | **30.904** | **0.036** | **0.271** |
| **Sex** | **0.262** | **1** | **0.262** | **60.772** | **0.017** | **0.244** |
| **Stress group * Sex** | **0.272** | **2** | **0.136** | **30.519** | **0.048** | **0.251** |
| **Error** | 0.812 | 21 | 0.039 |  |  |  |
| **Total** | 180.291 | 27 |  |  |  |  |
| **Corrected Total** | 10.669 | 26 |  |  |  |  |
| a. R Squared = 0.513 (Adjusted R Squared = 0.397) | | | | | | |

| **Pairwise Comparisons: Sex * Stress group** | | | | | | | |
| --- | --- | --- | --- | --- | --- | --- | --- |
| **Dependent Variable: CeA** | | | | | | | |
| **Stress group** | **(I) Sex** | **(J) Sex** | **Mean Difference (I-J)** | **Std. Error** | **Sig.** | **95% Confidence Interval** | |
|  |  |  |  |  |  | **LB** | **UB** |
| CTL | female | male | 0.042 | 0.139 | 0.764 | -0.247 | 0.332 |
| EASI | female | male | -0.186 | 0.132 | 0.173 | -0.460 | 0.088 |
| **LASI** | **female** | **male** | **-0.455*** | **0.127** | **0.002** | **-0.719** | **-0.191** |
| Based on estimated marginal means | | | | | | | |
| *. The mean difference is significant at the 0.05 level. | | | | | | | |

| **Pairwise Comparisons: Stress * Sex** | | | | | | | |
| --- | --- | --- | --- | --- | --- | --- | --- |
| **Dependent Variable: CeA** | | | | | | | |
| **Sex** | **(I) Stress group** | **(J) Stress group** | **Mean Difference (I-J)** | **Std. Error** | **Sig.** | **95% Confidence Interval** | |
|  |  |  |  |  |  | **LB** | **UB** |
| **female** | CTL | EASI | -0.116 | 0.139 | 0.413 | -0.405 | 0.173 |
|  |  | LASI | 0.240 | 0.139 | 0.098 | -0.049 | 0.530 |
|  | **EASI** | CTL | 0.116 | 0.139 | 0.413 | -0.173 | 0.405 |
|  |  | **LASI** | **0.357*** | **0.139** | **0.018** | **0.068** | **0.646** |
|  | LASI | CTL | -0.240 | 0.139 | 0.098 | -0.530 | 0.049 |
|  |  | EASI | -0.357* | 0.139 | 0.018 | -0.646 | -0.068 |
| **male** | **CTL** | **EASI** | **-0.345*** | **0.132** | **0.016** | **-0.619** | **-0.070** |
|  |  | LASI | -0.257 | 0.127 | 0.056 | -0.521 | 0.007 |
|  | EASI | CTL | 0.345* | 0.132 | 0.016 | 0.070 | 0.619 |
|  |  | LASI | 0.088 | 0.119 | 0.468 | -0.160 | 0.336 |
|  | LASI | CTL | 0.257 | 0.127 | 0.056 | -0.007 | 0.521 |
|  |  | EASI | -0.088 | 0.119 | 0.468 | -0.336 | 0.160 |
| Based on estimated marginal means | | | | | | | |
| *. The mean difference is significant at the 0.05 level. | | | | | | | |
